# On the Complexity of Resting State Spiking Activity in Monkey Motor Cortex

**DOI:** 10.1093/texcom/tgab033

**Published:** 2021-05-18

**Authors:** Paulina Anna Dąbrowska, Nicole Voges, Michael von Papen, Junji Ito, David Dahmen, Alexa Riehle, Thomas Brochier, Sonja Grün

**Affiliations:** 1 Institute of Neuroscience and Medicine (INM-6 and INM-10) and Institute for Advanced Simulation (IAS-6), Jülich Research Centre, Jülich 52425, Germany; 2 RWTH Aachen University, Aachen 52062, Germany; 3 Institut de Neurosciences de la Timone, CNRS-AMU, Marseille 13005, France; 4 Theoretical Systems Neurobiology, RWTH Aachen University, Aachen 52056, Germany

**Keywords:** balance, behavior-related spiking, dimensionality, macaque motor cortex, population activity

## Abstract

Resting state has been established as a classical paradigm of brain activity studies, mostly based on large-scale measurements such as functional magnetic resonance imaging or magneto- and electroencephalography. This term typically refers to a behavioral state characterized by the absence of any task or stimuli. The corresponding neuronal activity is often called idle or ongoing. Numerous modeling studies on spiking neural networks claim to mimic such idle states, but compare their results with task- or stimulus-driven experiments, or to results from experiments with anesthetized subjects. Both approaches might lead to misleading conclusions. To provide a proper basis for comparing physiological and simulated network dynamics, we characterize simultaneously recorded single neurons’ spiking activity in monkey motor cortex at rest and show the differences from spontaneous and task- or stimulus-induced movement conditions. We also distinguish between rest with open eyes and sleepy rest with eyes closed. The resting state with open eyes shows a significantly higher dimensionality, reduced firing rates, and less balance between population level excitation and inhibition than behavior-related states.

## Introduction

The resting state in behavioral studies is defined operationally as an experimental condition without imposed stimuli or other behaviorally salient events (Raichle 2009; Snyder and Raichle, 2012). It has become a classical paradigm for experiments involving *large-scale* measurements of brain activity like functional magnetic resonance imaging (fMRI) and magneto- and electroencephalography in humans and monkeys (Vincent *et al.* 2007; Raichle, 2009; Deco *et al.* 2011; Snyder and Raichle, 2012; Baker *et al.* 2014), but it has also been studied on the *level of single brain areas*. Here, the spontaneous activity is referred to as ongoing, intrinsic, baseline, or resting state activity and can be studied by means of, for example, optical imaging combined with single electrode recordings (Arieli *et al.* 1996; Tsodyks *et al.* 1999; Kenet *et al.* 2003). Such data, collected under anesthesia, were used to investigate the variability in evoked cortical responses (Arieli *et al.* 1996), the switching of cortical states (Tsodyks *et al.* 1999), and their link to the underlying functional architecture (Kenet *et al.* 2003). With our study, we aim to characterize the resting state on yet another spatio-temporal scale, namely on the *scale of simulateneous single unit (SU) spiking activity* recorded in the motor cortex of *awake* macaque monkeys.

Spiking activity in monkey motor cortex has been studied extensively during arm movements, which give rise to an increased average neuronal firing compared with wait (Nawrot *et al.* 2008; Rickert *et al.* 2009; Riehle *et al.* 2018). On a SU level, direction-specific neuronal subpopulations encode the movement direction by firing rate (FR) modulations (Georgopoulos *et al.* 1986; Rickert *et al.* 2009). These and other studies also investigated the spike time irregularity and the spike count variability in monkey motor cortex during various behavioral epochs: movements have been related to a lower spike count variability across trials (Rickert *et al.* 2009; Churchland *et al.* 2010; Riehle *et al.* 2018) and to a higher spike time irregularity (Davies *et al.* 2006; Riehle *et al.* 2018) compared with wait or preparatory behavior without movements. However, the resting state we analyze in this study is conceptually distinct from waiting or preparatory epochs: there is no task to prepare for and no signal to be anticipated. It is a state without any particular expectations or dispositions.

What is the interest of resting state studies? A major conclusion of large-scale measurements is that the spontaneous activity can be characterized as a sequence of re-occurring spatio-temporal patterns of activation or deactivation resembling task-evoked activity (Fox and Raichle 2007; Vincent *et al.* 2007; Heuvel and Hulshoff Pol 2010), a special example being the so-called default mode network of brain regions with strong deactivation during cognitive tasks (Biswal *et al.* 1995; Raichle 2009; Deco *et al.* 2011). Likewise, the aim of resting state studies on the level of single neurons is a comparative characterization of spiking activity and its coordination between neurons at rest in the awake animal. Such a characterization serves not only to explain the variability in cortical responses or to differentiate between different states (Arieli *et al.* 1996; Tsodyks *et al.* 1999; Churchland *et al.* 2010; Riehle *et al.* 2018), but it also provides data to validate realistic models of cortical networks (Markram *et al.* 2015; Schmidt *et al.* 2018a, 2018b; Billeh *et al.* 2020) Such models are derived from anatomical and electrophysiological data and aim to mimic brain dynamics and function down to the level of individual neuron activities. A prerequisite to understand mechanisms underlying task-related dynamics and function in these models is, however, to first realistically emulate the idle state and intrinsically generated dynamics (Vreeswijk and Sompolinsky 1996, 1998; Brunel 2000; ; Kumar *et al.* 2008; Voges and Perrinet 2010; Potjans and Diesmann 2014; Dahmen *et al.* 2019). Therefore, it is important that benchmark data during resting state on the level of single-neuron spiking activities is available.

There are computational frameworks to be used for a quantitative comparison or, ultimately, validation of such spiking neural network models against experiments (Gutzen *et al.* 2018), but data on SU activity in the awake resting-state condition is still lacking. As a consequence, network models are often compared with data collected from anesthetized subjects (Kenet *et al.* 2003; Renart *et al.* 2010) or in behavioral experiments, where tasks or stimuli lead to transient deviations from the resting state measurements such as, for example, increasing average FRs during movements (Georgopoulos *et al.* 1986; Kaufman *et al.* 2013; Riehle *et al.* 1997; 2018). Therefore, to provide a suitable reference for the validation of spiking neural network models on the single neuron level, we here present an analysis of parallel spiking activity in awake macaque monkeys at rest.

The aim of this study is a detailed characterization of the spiking activity at rest compared with task-induced and spontaneous movements. To this end, we recorded the ongoing activity with a 4$\times $4 mm$^2$ 100 electrode Utah Array (Blackrock Microsystems, Salt Lake City, UT, USA) situated in the hand-movement area of macaque motor cortex. We performed 2 types of experiments:

1. During resting state (REST) experiments, we recorded the neuronal activity of 2 monkeys seated in a chair with no task or stimulation. The spontaneous behavior was then classified into periods of rest, movements, and sleepy rest (eyes closed).
2. Reach-to-grasp (R2G) experiments (Torre *et al.* 2016; Riehle *et al.* 2013, 2018; Brochier *et al.* 2018) provide well-defined periods of task-related movements and task-imposed waiting. The latter behavior is similar to rest but contains a mental preparation task.

We ask if a distinction between spontaneous (resting and nonresting) and task-evoked (R2G) neuronal dynamics is expressed on the level of SU and network spiking activity. More specifically, we also ask if certain features of the neuronal firing during pure resting periods allow for a differentiation from spontaneous and task-induced movements, preparatory periods, or sleepiness. Contrary to this expectation, the motor system may show invariants, that is, statistical properties of the neuronal spiking that do not change with respect to different behavioral epochs. Although such comparisons have been performed on the level of local field potential (LFP) recordings, for example, the investigation of behavior-related frequency modulations (Engel and Fries 2010; Kilavik *et al.* 2013), to our knowledge this is the first study to perform such comparison on the level of spiking activity.

In the following, we first detail how we performed the segmentation of REST recordings according to behavior, and then explored the activity of single neurons in different behavioral states. To investigate if there are comparable neuronal activity states in task-related data, we performed similar analyses for the R2G data. Apart from the SU dynamics, we also focused on network properties of the neuronal activities: We evaluated pairwise covariances (COVs), dimensionality of rate activities, and excitatory-inhibitory balance in the different behavioral states of both REST and R2G. The comparison to the R2G data enabled us to identify systematic network state changes which are less pronounced in REST.

## Materials and Methods

We first describe the 2 types of experimental recordings analyzed in this paper: REST and R2G data. Then, we explain the experimental procedure and the preprocessing of all data types with a particular focus on the REST recordings and their behavioral classification. Finally, the measures calculated to characterize different behavioral states are listed and explained.

### Experimental Paradigm and Recordings

Two adult macaque monkeys (*Macaca mulatta*), a female (monkey E), and a male (monkey N), participated in 2 distinct behavioral experiments: resting state (REST) and reach-to-grasp (R2G). The animal procedures were approved by the local ethical committee (C2EA 71; authorization A1/10/12) and conformed to the European and French government regulations. Monkeys were chronically implanted with a 4 $\times $ 4 mm$^2$ 100 electrode Utah Array (Blackrock Microsystems, Salt Lake City, UT, USA) situated in the hand-movement area of the motor cortex. However, because of unavoidable imprecision of the array placement, a few electrodes at its edges might have been placed in the premotor cortex. Spiking activity and LFP were recorded continuously during an experimental session, with sampling frequency of 30 kHz. The LFP signal was then down-sampled to 1 kHz. Details on surgery, recordings, and spike sorting, as well as on the R2G settings are described in Brochier *et al.*  (2018), Riehle *et al.* (2013, 2018).

During a REST session, the monkey was seated, but not immobilized, in a primate chair. It was only fastened to the chair with a collar. The chair was positioned so as to prohibit the animal from reaching any objects. There was neither a particular stimulus nor any task, the monkey was free to look around and move spontaneously. In addition to the registration of brain activity, the monkey’s behavior was video recorded and synchronized with the electrophysiology recording. For each monkey, 2 such sessions were recorded and lasted approximately 15 min for monkey N and 20 min for monkey E.

**Figure 1:**
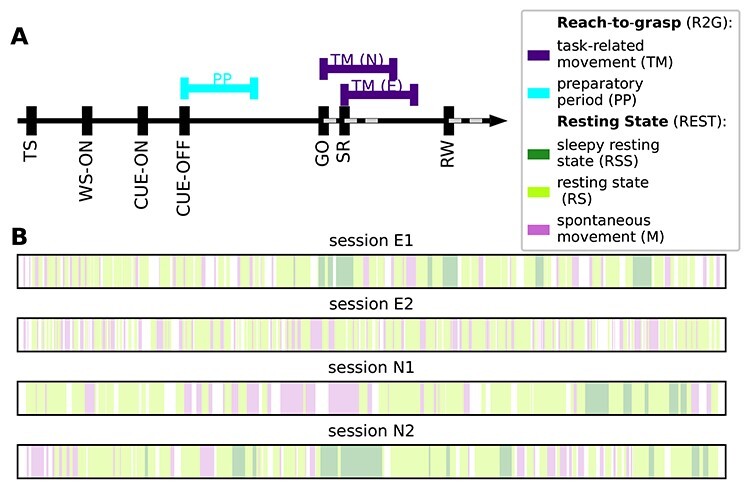
Behavioral segmentation in REST and R2G recordings. (*A)* Order and timing of events within a single trial of an R2G session. Colored lines above the time axis indicate time intervals considered for the analysis: preparatory period (PP, cyan) and task-induced movements (TM, purple). SR indicates the switch-release event—beginning of the hand movement. PP was defined as [CUE-OFF, CUE-OFF+500 ms], and TM as [SR, SR+500 ms] for monkey E, and [SR-150 ms, SR+350 ms] for monkey N (different for the 2 monkeys due to differences in performance speed). (*B*) Order and relative duration of the behavioral states defined within each REST session (single second precision): resting state (RS, light green) represents no movements and eyes open, sleepy rest (RSS, dark green) represents no movements and eyes (half-)closed, spontaneous movements (M, pink) represent movements of the whole body and limbs.

In the R2G experiments, monkeys were trained to perform an instructed delayed reach-to-grasp task to obtain a reward, see Figure 1*A*. The monkey had to self-initiate a trial by closing a switch (TS). After 800 ms, a CUE-ON signal provided some task-related information. After another 300 ms, the CUE signal was switched off which defined the start of the preparatory delay, during which the monkey was required to sit still. One second after the CUE-OFF, a GO signal provided the complementary task-related information and indicated the monkey to start moving. The monkey had to release the switch (SR) and reach to the target. After grasping the object, the monkey had to pull and hold it for 500 ms to obtain the reward (RW). Brain activity was recorded together with time stamps of all events within a trial.

Typically, a REST recording was performed subsequent to an R2G recording session. Only the second session of monkey E (E2) was recorded directly before an R2G session, which is probably the reason for the missing RSS intervals. The monkey was rather twitchy, impatiently waiting for the R2G tasks, likely because R2G experiments include a reward, whereas there was no reward during REST recordings.

### Behavioral Segmentation

Based on video recordings, each REST session was segmented into intervals of several behavioral states. Three states were defined with single-second precision as follows: resting state (RS)—no movements and eyes open; sleepy resting state (RSS)—no movements and eyes (half-)closed; and spontaneous movements (M)—accounting for movements of the whole body and limbs, see Figure 1*B*.

If a movement or a closed-eyes interval began during a particular second, this whole second was classified as M or RSS, respectively. Eye movements and minor head movements were allowed during RS. All other types of behavior (e.g., strong isolated head movements) and periods for which it was not possible to clearly classify the monkey’s behavior (e.g., due to a lack of visibility) were considered as unclassified and excluded from analyses. To increase the reliability of classification, behavioral scoring for each session was performed by 2 independent observers, and the results were merged later. In case of any conflicts, the parts of the video in question were rewatched and reclassified upon agreement.

**Figure 2:**
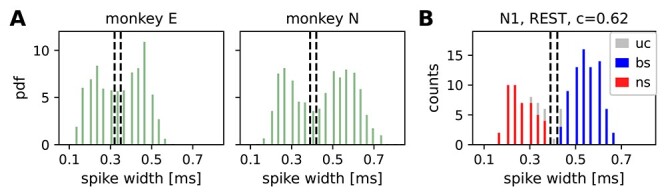
Separation between broad-spiking (bs) and narrow-spiking (ns) SUs. (*A*) Spike-width distributions calculated from the average spike widths of all REST and R2G recording sessions for monkey E and monkey N. The 2 vertical lines indicate the thresholds for ns (0.33 ms and 0.4 ms for monkeys E and N, respectively) and bs units (0.34 ms and 0.41 ms for monkeys E and N, respectively). (*B*) Exemplary separation between ns (red) and bs (blue) units for the first REST recording of monkey N (N1). SUs between the thresholds are left unclassified (uc, gray), as well as all SUs with a consistency smaller than 62%.

Behavior classification in R2G recordings was based on the events registered throughout the experiment, see Materials and  Methods: Experimental Paradigm and Recordings. Two periods within a trial (see Fig. 1*A*) were considered: preparatory period (PP), defined as 500 ms after the CUE-OFF (first half of the preparatory delay, no movements), and task-related movement (TM)—500 ms after SR, including grasping. Because of differences in performance speed of each monkey, this period was defined as: [SR, SR+500 ms] for monkey E, and [SR-150 ms, SR+350 ms] for monkey N. All successful trials were used.

Since the amount of data strongly differs between behavioral states in REST, we used data slices of equal length, mostly 3 s slices, to have comparable statistics. The choice of slice length represents a compromise between different factors: 1) the slice length cannot exceed the typical duration of each behavior (shortest for movement), 2) the slice length should be as long as possible for reliable estimation of COVs within each slice,and 3) to average across slices, we need as many slices as possible. Following these arguments, each behavioral segment was cut into as many continuous slices as possible. For example, if a REST segment was 7 s long, it was separated into 2 slices of 3 s and the remaining 1 s was not considered for the analysis. In the R2G data, the slice length for the 2 behavioral states was 0.5 s by definition, see above. When directly comparing REST and R2G data, we either considered 0.5 s slices for the REST data (comparison of firing statistics) or we concatenated six 0.5 s slices of the R2G data to 3 s slices (analysis of COVs and balance).

### Preprocessing

The waveforms of potential spikes were sorted into the SUs offline and separately on each electrode using the Plexon Offline Spike Sorter (version 3.3, Plexon Inc., Dallas, TX, USA), see Riehle *et al.*  (2018). Synchrofacts, that is, spike-like synchronous events across multiple electrodes at the sampling resolution of the recording system (1/30 ms) (Torre *et al.* 2016), were then removed. Sorted units were separated into broad-spiking (bs) and narrow-spiking (ns) SUs representing putative excitatory and inhibitory neurons, respectively. The separation was achieved by thresholding the spike-widths distribution (Bartho *et al.* 2004; Kaufman *et al.* 2010, 2013; Peyrache *et al.* 2012; Dehghani *et al.* 2016) in the following way. For a given monkey, average waveforms from all SUs recorded in all considered sessions (REST and R2G) were collected. Based on the distribution of spike-widths (time interval between trough and peak of a waveform), thresholds for “broadness” and “narrowness” were chosen such that the values in the middle of the distribution remained unclassified (Fig. 2). For monkey N, spikes with a width shorter than 0.4 ms were considered to be narrow (ns SUs, putative inhibitory neurons), whereas spikes longer than 0.41 ms were considered to be broad (bs SUs, putative excitatory neurons). For monkey E, spikes narrower than 0.33 ms were considered as ns SUs and spikes broader than 0.34 ms were considered as bs SUs. The difference between monkeys was due to different parameters of causal Butterworth filters applied prior to spike detection: 250–5000 Hz with both high-pass and low-pass order 2 for monkey N and 250–7500 Hz with high-pass order 4 and low-pass order 3 for monkey E.

Next, a 2-step classification was performed. For a given session, the thresholds were applied to the averaged SU waveforms (first preliminary classification). Secondly, the single waveforms of all SUs were thresholded and a consistency measure $c$ was calculated per SU: the percentage of SU single waveforms preliminarily classified as broad. If $c>0.5$, a SU was classified as bs; if $c<0.5$, a SU was classified as ns (second preliminary classification). Typically, these 2 classifications yielded inconsistent results for some SU, for example, a SU with majority of spikes slightly narrower than 0.4 ms has been classified (based on its average waveform) as bs SU. During an iterative procedure, we increased the minimal required consistency until there were no more contradictions in the results of both preliminary classifications. SUs with high enough consistency were then declared classified as putative excitatory or inhibitory. SUs whose mean waveform’s widths fell between two thresholds or whose consistency was too low were declared unclassified.

Only SUs with signal-to-noise ratio (SNR, see Hatsopoulos *et al.*  2004) of at least 2.5 (cf. Torre *et al.*  2016; Brochier *et al.*  2018; Riehle *et al.*  2018), and a minimal average FR of 1 Hz were considered for the analysis to ensure enough and clean data for valid statistics. Critical considerations on our assumptions, in particular the approach to separate ns and bs SUs, are given in Discussion: Influence of Preprocessing and Critical  Assumptions.

Table 1 lists all single recording sessions for both REST and R2G experiments. It provides information about the number of SUs (separated into putative excitatory/bs and putative inhibitory/ns) and the number of data slices (3 or 0.5 s long in REST) or trials (R2G), in the different behavioral states. Thus, in total, we have 2 sessions of 15–20 min from each monkey during REST, and 6/5 sessions (of similar durations) of monkey E/N during R2G. This results in 627 R2G trials of monkey E and 635 trials of monkey N. These were compared with the following numbers of data segments of 0.5 s during REST: 232, 2028, and 508 segments of RSS, RS, and M, respectively, for monkey E and 372, 1676, and 492 segments for monkey N.

**Table 1:** List of all considered experimental recordings

REST session	#slices: 3 s (0.5 s)			#SUs (#bs, #ns)
	RSS	RS	M	
e170103-002 (E1)	40 (232)	196 (1058)	43 (200)	115 (56, 50)
e170131-002 (E2)	0	189 (970)	67 (308)	133 (67, 56)
i140701-004 (N1)	20 (114)	151 (840)	58 (312)	130 (76, 45)
i140615-002 (N2)	45 (258)	156 (836)	36 (180)	154 (78, 62)
R2G session		#trials		#SUs (#bs, #ns)
e161212-002		108		129 ( 50, 55)
e161215-001		102		89 ( 39, 36)
e161220-001		114		96 ( 45, 34)
e161222-002		102		118 ( 58, 41)
e170105-002		101		116 ( 61, 44)
e170106-001		100		113 ( 56, 42)
i140613-001		93		137 ( 71, 56)
i140616-001		130		152 ( 75, 60)
i140617-001		129		155 ( 84, 59)
i140703-001		142		142 ( 84, 46)
i140704-001		141		124 ( 70, 43)

Note: Session names (first column) starting with “e” refer to monkey E, and with “i” to monkey N. Throughout the manuscript the REST sessions are referred to as E1, E2, N1, and N2. Each R2G trial yields 1 PP and 1 TM period, equally long (0.5 s each).

#### LFP Spectra

The spectral density of the LFP in different behavioral states in REST and R2G data was estimated with Welch’s method provided by Elephant (https://python-elephant.org). We considered 3 s slices for the REST and 0.5 s slices for the R2G data. The spectra were obtained by averaging over single spectra from state-specific slices of all respective recordings. We used a Hanning window of 1 s and an overlap of 50% for the REST data, whereas the R2G spectra were estimated with a Hanning window of 0.3 s with an overlap of 50%. Additionally, an artifact in session N1—high-amplitude synchronous peak on all recording channels—was removed: it was replaced by the average of the remaining signal.

### Data Analysis

To characterize and compare different behavioral states, we employed a set of analysis tools. We quantified the correlation between neuronal firing and behavior, and we calculated the dimensionality of population spiking activity and the balance between time-resolved putative excitatory and inhibitory population counts.

Preprocessing and data analyses were performed in Python, version 2.7, with the Elephant package (https://python-elephant.org). Since the distributions of our measures were typically non-Gaussian, the significance of differences between them was assessed via Kruskal–Wallis tests for multivariate comparisons (KW, nonparametric alternative to a one-way analysis of variance), with significance level $\alpha =0.001$. Multiple comparisons were corrected for with a Bonferroni–Holm correction.

For visualizations of distributions obtained for different behavioral states, we used notched box plots. The line in the center of each box represents the median, box’s area represents the interquartile range, and the whiskers indicate minimum and maximum of the distribution (outliers excluded).

#### Behavioral Correlation

For each REST session, we defined a state vector based on the behavioral segmentation, see Materials and Methods: Behavioral  Segmentation. Each element of the state vector represented the behavioral state of a 1 s slice of the recording and was set to $-1$ for RS, to 1 for M, and zero otherwise. To assess the relation between the activity of each SU and monkey’s behavior, the average FR in each 1 s slice was correlated (Spearman rank correlation) with the corresponding element of the state vector. Only pairs of entries in which the state vector was different from zero were considered. This procedure resulted in a value, which we called behavioral correlation: BC $\in $ [$-1,1$], and the corresponding $P$ value (indicating statistical significance if $P<0.001$ after Bonferroni–Holm correction for the number of SUs) for each SU. Positive BC indicated that the FR increased during movements or decreased during rest, and vice versa for negative BC. We investigated the distributions of BC values separated between ns and bs SUs.

For a substantiation of these results, we additionally compared the FRs during all 3 behavioral states defined in REST, again separately for each SU: We applied Kruskal–Wallis test on FR values obtained in all 3 s data slices to check for significant differences between M, RS, and RSS states. Note that this method does not provide any quantification of the strength of the correlation as represented by BC.

For both tests described above, we calculated the percentage of SUs that changed their FR significantly with changes in the behavioral states. This percentage was computed for all SUs and also separately for ns and bs neurons.

Furthermore, we performed pairwise comparisons between 3 behavioral states per SU (again with Kruskal–Wallis tests and in 3 s data slices), asking specifically for a significant increase or decrease when comparing the FRs in any 2 states. We then computed the percentages of all SUs, which either significantly increased or decreased their FR in one state with respect to the other.

**Figure 3:**
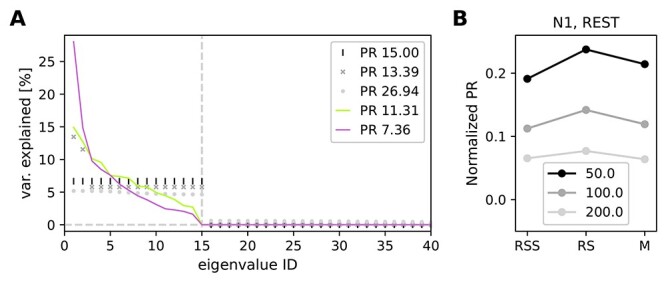
Participation ratio (PR) to characterize the dimensionality. (*A*) Sketch showing relation between the eigenvalue spectrum and PR. If the first $N$ out of 100 eigenvalues explain equal amounts of variance and the rest vanishes, the PR equals $N$ (black vertical lines for $N=15$). If a few eigenvalues are much higher than the others, the resulting PR decreases (dark gray crosses). If, on the contrary, a uniformly distributed random value is added to each eigenvalue from the first case, the calculated PR becomes higher (light gray circles). Experimental data are typically a mixture of the second and third case. Continuous traces show exemplary results for a single 3 s data slice of RS (green) and M (pink) in session N1. (*B*) PR was calculated with different bin sizes (50, 100, and 200 ms) in exemplary session N1. PR values were normalized by the number of SUs recorded in this session.

#### Neuronal Firing in REST and R2G States

To compare the SU firing properties in behavioral states from different experiments, we used 0.5-s-long slices of both REST and R2G recordings. In REST, the single seconds at the transitions from one state to another were excluded. For each time slice of each SU, we estimated the average FR and the local coefficient of variation (CV2) (Ponce-Alvarez *et al.* 2010; Riehle *et al.* 2018; Voges and Perrinet 2010), and per SU across slices the Fano factor (FF) (Nawrot *et al.* 2008; Nawrot 2010; Riehle *et al.* 2018), which describes the variability in SU spike counts across trials (R2G) or time slices (REST). For the REST recordings, we also calculated the commonly used coefficient of variation (CV) (Shinomoto *et al.* 2003; Ponce-Alvarez *et al.* 2010; Voges and Perrinet 2010), shown in the additional figure in Supplement 1. CV and CV2 are based on the inter-spike-interval distribution of a SU: they characterize the (ir-)regularity in neuronal firing. The CV2 corrects for transient firing rate changes which yield inappropriately high CV values (Ponce-Alvarez *et al.* 2010; Voges and Perrinet 2010).

We compared the FR and CV2 values obtained for each SU within each slice of RSS, RS, M, TM, and PP states in 2 different ways. On the one hand, we averaged over time slices/trials to represent the variability with respect to SUs. On the other hand, we averaged the results obtained for each data slice/trial over SUs in order to analyze the variability of our measures in time. The significance of the differences between the behavioral states was assessed with a Kruskal–Wallis test including a Bonferroni–Holm correction, both when comparing all 5 states and in pairwise comparisons.

#### Covariance and Dimensionality

To measure the joint variability in rate modulation, we calculated the pairwise spike count covariance (COV) (Cohen and Kohn  2011; Dahmen *et al.*  2019). REST data were cut, and R2G data were concatenated into 3 s slices (state-resolved) and binned into 100 ms intervals. The bin size of 100 ms was a compromise between obtaining enough bins to calculate COV values (given a slice length of 3 s), considering enough spikes for reliable estimation of COV, and using a time scale appropriate for the examination of rate modulations. Correspondingly, the R2G data from 6 consecutive trials contributed to a single COV value.

The COV between spike trains $i$ and $j$ was defined as:

(1) \begin{align*}& \textrm{COV}(i,j) = \frac{\langle \mathbf{b_i} - m_i, \mathbf{b_j} - m_j \rangle} {l-1}, \end{align*}

with $\mathbf{b_i}$ and $\mathbf{b_j}$—binned spike trains, $m_i$ and $m_j$ being their mean values, $l$ the number of bins, and $\langle \mathbf{x},\mathbf{y} \rangle $ the scalar product of vectors $\mathbf{x}$ and $\mathbf{y}$. Thus, for each 3 s slice of a particular state, we obtained a COV matrix $\mathbf{M}\in \mathbb{R}^{N_{SU} \times N_{SU}}$ with $N_{SU}$—number of SUs.

Based on the COV matrices, we calculated the participation ratio (PR) to characterize the dimensionality of activity in different behavioral states, see Mazzucato *et al.*  (2016), Gao *et al.*  (2017). Eigenvalue decomposition of COV matrix $\mathbf{M}$ yields $N_{SU}$ eigenvalues $\lambda $ with corresponding eigenvectors $\mathbf{\nu }$, such that $\mathbf{M} \mathbf{\nu }_i = \lambda _i \mathbf{\nu }_i$. The eigenvalues were used to calculate the PR of the neuronal dynamics:

(2) \begin{align*}& \textrm{PR} = \frac{(\sum_i\lambda_i)^2}{\sum_i\lambda_i^2}. \end{align*}

The PR thus quantifies how many eigenvectors are necessary to explain a significant part of variance in dynamics described by $\mathbf{M}$.

The PR is low if most of the variability is captured by the first few eigenvectors. A large PR indicates that many eigenvectors are necessary to capture the dynamics—a sign of high complexity, see Figure 3*A*. In order to test the robustness of our results, we performed our analysis with different bin sizes. The result is shown in Figure 3*B*. Here, all bin sizes revealed the same PR-dependent ordering of behaviors. This suggests that our results are robust to the choice of bin size.

The value of the PR depends on the number of SUs present in the analysis: It can take values $1\leq $ PR $\leq N_\textrm{SU}$. Additionally, the relation between the PR and $N_\textrm{SU}$ is generally not linear (see Supplement 3). In order to make the PRs comparable across recording sessions with different numbers of SUs, we computed the PR always using only 89 SUs, which is the number of SUs in the session with the smallest number of SUs (i.e., session e161215-001). To avoid any bias in PR values due to a specific choice of SUs, for each 3 s long data slice a random subsampling of 89 SUs for PR computation was repeated 100 times, and the obtained PRs were averaged over the repetitions. Thus, we get 1 PR value per data slice, in the range [1, 89].

#### Balance between Putative Excitatory and Inhibitory Firing

The balance between putative excitatory (bs SUs) and inhibitory (ns SUs) population firing was examined similarly to the procedure proposed in Dehghani *et al.*  (2016). In the first place, we examined the differences between z-scored population activities at various time scales (bin sizes from 1 ms to 10 s) across the whole recording (no state-specific slices). Obtained values were generally close to zero, indicating balanced activity. However, for bin sizes $\ge $30 ms, we observed transient deviations from perfect balance. This means that the difference between the ns and bs population activities becomes occasionally large relative to the magnitude of their fluctuations on such time scales. To compare the level of balance in different behaviors, we selected the bin size of 100 ms, but similar results can be obtained with bin sizes up to 500 ms. For each REST and R2G session of a given monkey, we binned the 3 s time slices (concatenated from 6 consecutive trials of 0.5 s for R2G data) into 100 ms bins (in the same manner as for the dimensionality calculation). Next, we applied 2 methods to quantify the balance between population activities.

Firstly, we z-scored the population activities, using the respective mean and standard deviation of the whole recording (not state-specific). Then, we calculated, separately for each state, the difference between the z-scored bs and ns population activity of each 100 ms bin in each time slice: If this difference was close to zero, that is, if pooled ns and bs spike counts were nearly identical, the network activity was called balanced. A negative value indicated a domination of ns activity, whereas a positive value meant that the bs activity was higher.

Secondly, we calculated the Spearman rank correlation between raw bs and ns population activities across 100 ms bins within each 3 s time data slice: The higher the correlation $\rho (\textrm{bs},\textrm{ns})$, the more strict the balancing between the ns and bs populations (cf. Renart *et al.*  2010; Tetzlaff *et al.*  2012).

To investigate the relationship between balance and dimensionality, we calculated the Spearman rank correlation between $\rho (\textrm{bs},\textrm{ns})$ and PR for each monkey, pooled over all REST and R2G sessions, respectively.

## Results

We aim to determine in what regards spiking activity during rest is distinct from other behavioral states like spontaneous movements, sleepiness, movement preparation, or task-induced grasping. To this end, we first clarify that the behavioral segmentation is meaningful in terms of neuronal activity on 2 different scales. On the mesoscopic scale, which incorporates the collective behavior of neurons, we show that the LFP spectra differ considerably across states. On the microscopic scale, we show that SU firing is clearly correlated to the monkeys’ behavior. We then examine the relations between behavior, spiking activity dimensionality and excitatory-inhibitory balance.

### Behavioral Segmentation

For each REST session, the behavioral segmentation provides data segments of the following 3 states: resting state (RS)—no movements and eyes open; sleepy resting state (RSS)—no movements and eyes (half-)closed; spontaneous movements (M)—movements of the whole body and/or limbs (cf. Fig. 1*B*). For R2G recordings, we extracted 2 behavioral states defined with respect to trial events (Riehle *et al.* 2018): the preparatory period (PP) and task-induced movements (TM). Table 1 in Materials and Methods lists all recording sessions for both REST and R2G experiments together with the resulting number of SUs and data segments.

**Figure 4:**
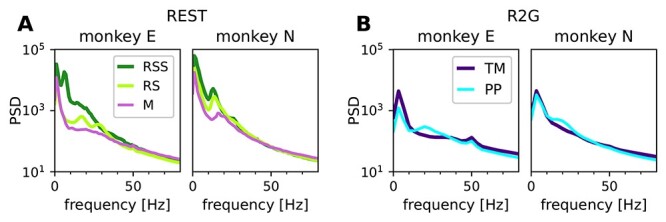
Power spectral density of LFP in different behavioral states. Panels in **A** pertain to REST, panels in **B** to R2G, left for monkey E and right for monkey N, respectively. States are defined in Figure 1*A* and *B*. The peak at 50 Hz in the R2G spectra is an artifact (line frequency) and was not considered.

The segmentation based on video recordings is substantiated by comparison of the LFP spectra in the above-defined states (Fig. 4). The relationship between LFP and behavior has been shown in several studies, for example, Pfurtscheller and Aranibar  (1979), Fontanini and Katz  (2008), Engel and Fries (2010), Takahashi *et al.*  (2011), Kilavik *et al.*  (2013). Beta oscillations ($\approx $13 to 30 Hz) have been linked to states of general arousal, movement preparation, or postural maintenance (Baker *et al.* 1999; Kilavik *et al.* 2012) and are typically suppressed during active movement (Pfurtscheller and Aranibar 1979).

In our data, the awake, no-movement periods RS and PP show peaks in the range from $\approx $10 to $\approx $30 Hz (alpha/beta range), the peak in PP occurs for a higher frequency than in RS (Fig. 4). In both monkeys, the movement periods M and TM contain more power compared with other states in frequencies above $\approx $50 Hz (gamma), whereas beta power is reduced. However, the spectrum during sleepiness RSS differs between monkeys. In monkey E, RSS seems to be a distinct physiological state: it shows strong slow oscillations, as to be expected (Gervasoni *et al.* 2004; Fontanini and Katz 2008) for a sleepy version of RS. In monkey N, however, the spectra during RSS are more similar to RS, but still with more power in the lower frequency bands.

### Relation between Neuronal Firing Rates and Behavior

A prerequisite for the following analyses is to formalize a relationship between neuronal spiking activity and the behavioral states of a monkey. Therefore, we quantified the correlation between SU firing and the behavioral states.

**Figure 5:**
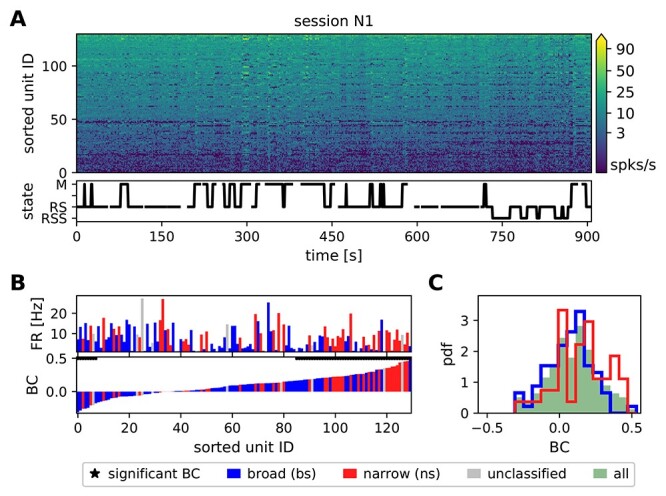
The correlation between SU firing and behavior for 1 REST session of monkey N. (*A*) Time- and population-resolved firing (spikes/s). SUs are sorted according to average FRs in increasing order from bottom to top. The behavioral state of the monkey is shown below. The time resolution is 1 s. Empty spaces denote periods of unclassified behavior, vertical lines indicate transitions between identified states. (*B*) Comparison of average FRs (top row) and behavioral correlation values (bottom row, only M and RS states are taken into account). The SUs in both diagrams are sorted according to increasing values of BC. Blue bars indicate bs (putative excitatory) and red ns (putative inhibitory) SUs, gray indicates unclassified units. Black stars above the BC bars indicate significant correlations. (*C*) Distributions of BC values. In this recording session the difference between the ns (red) and the bs (blue) distribution is significant.

Figure 5*A* shows the time-resolved FR of all recorded SUs in 1 REST session (N1) (see Materials and Methods: Behavioral  Correlation). They change in time and are variable across SUs, which is true for all REST sessions. The FRs range from 0 up to $\approx $100 spikes per second. Some SUs exhibit a constant firing (not visible by eye), for example, unit 127 in Figure 5A with a small (in relation to the mean value) standard deviation, FR $=25.29\pm 6.46$. The firing of other SUs changes considerably over time, for example, unit 17 with a relatively large standard deviation FR $=1.74\pm 3.53$.

To examine this variability with respect to the behavior of the monkey, we defined a behavioral state vector (bottom panel of Fig. 5*A*). Its entries represent the behavioral states: the value is set to +1 if there are movements (M) and $-1$ if the monkey is at rest (RS), and for the following analysis all other states are not taken into account. The bottom row of Figure 5*B* shows the value of correlation between the state vector and the FR (in 1 s bins) of each of the SUs, termed as behavioral correlation (BC, see Materials and Methods: Behavioral Correlation), ordered from minimum to maximum of the obtained BC value. The bars in the panel above are ordered identically and show the FRs of the corresponding SUs, averaged across the whole recording. Most SUs increase their FR during M (mostly on the right side of the panel), many of them significantly ($\textrm{BC}>0.17$, $P<0.001$). A much smaller set of SUs increases their firing during RS ($\textrm{BC}<-0.17$), seen mostly on the left side of the panel. This asymmetry between the 2 states is reflected by the positive average BC in all 4 REST sessions (Table 2 column 3). The second column of Table 2 lists the percentage of SUs with significant BC, for all sessions. They range from 40.8 to 66.9%, however, neither the sign nor the amount of the behavioral correlation can be reliably predicted from the average FR: Both SUs with very high or very low mean FR show negative, positive and close to zero BC values. This is also indicated by the insignificant correlation between FR and BC (Table 2 column 4): $\rho _{\text{BC,FR}}$. Yet, the consistently negative $\rho _{\text{BC,FR}}$ values suggest that SUs with lower FRs tend to be more sensitive to behavior than highly active ones (the lower the mean FR, the higher the mean BC).

**Table 2:** Behavioral correlation for all REST sessions

Session	% SUs$_{\textrm{BC}}$ (bs, ns)	mean BC (bs, ns)	$\rho _{\text{BC,FR}}$	% SUs$_{\textrm{KW}}$ (bs, ns)
E1	53.9 (50, 60)	0.13 (0.14, 0.13)	–0.19, $P$ = 0.03	54.7 (51.7, 58)
E2	66.9 (64.2, 71.4)	0.14 (0.14, 0.15)	–0.09, $P$ = 0.3	76.9 (79.1, 76.8)
N1	40.8 (32.9, 53.3)	0.10 (0.07, 0.15)	–0.13, $P$ = 0.2	48.1 (39, 62.2)
N2	46.8 (39.7, 54.8)	0.12 (0.09, 0.14)	–0.04, $P$ = 0.7	43.5 (39.7, 50)

Note: The second column gives the percentage of SUs that show a significant behavioral correlation (BC, $P<0.001$), the third column gives BC averages, and the fourth column the Spearman rank correlation between average single unit FR and BC ($\rho _{\text{BC,FR}}$). Column 5 lists the percentages of SUs that change their firing significantly ($P<0.001$) with the behavioral state, obtained with a Kruskal–Wallis test on M, RS, and RSS. The numbers in brackets indicate the values obtained when separating between bs (first entry) and ns (second entry) SUs. 1 s resolution.

In order to include the RSS state (in addition to M & RS) in the correlation of neuronal activity and behavioral states, we performed a Kruskal–Wallis test, per SU, for differences in FR among the 3 behavioral states. This provides information about the significance, but no quantification of the strength of the correlation. The obtained percentage of significantly correlated SUs ($P<0.001$) ranges from 55 to 77% in monkey E and from 44 to 48% in monkey N (last column of Table 2). Thus, we find a clear inter-relation of the behavioral state and the neuronal activity.

Figure 5*C* shows the distribution of the BC values in session N1 for all SUs (green shaded area), and for SUs separated into putative excitatory/bs SUs and putative inhibitory/ns SUs (blue and red lines, respectively). In this session, we find a significant difference between the distributions for ns and bs SUs. However, this could not be substantiated in the data from other recording sessions, indicating that the neuron type does not determine the strength of correlation with behavior. Still, the firing of putative inhibitory as compared with excitatory neurons seems to be more related to behavioral states. This is indicated by higher percentages of significantly correlated ns than bs SUs (cf. Table 2 column 2 and 5), particularly in monkey N, see also the higher mean BC of ns units in monkey N (cf. Table 2 column 3).

To examine in more detail behavior-related modulations of average FR, we performed a set of pairwise comparisons between behavioral states per SU (using 3 s slices, see Materials and Methods: Behavioral Correlation). Table 3 summarizes the results by listing the percentages of SUs that significantly change their FR with respect to behavior. We observe that $\approx $34 to 67% of the SUs show significantly higher FRs during M as compared with RS, but still, 5–11% of SUs show significantly higher FRs during RS (second and fifth column in Table 3). Correspondingly, the percentages for RSS versus M show a similar tendency ($\approx $25 to 48% and 2 to 8%, respectively, column 3 and 6). This confirms the results obtained so far, that there are mostly lower FRs during rest (RS and RSS) than during movement (M).

**Table 3:** Pairwise comparisons of SU firing rates in different states

Session	RS < M	RSS < M	RS < RSS	RS > M	RSS > M	RS > RSS
E1	37.6	24.8	18.8	7.7	7.7	3.8
E2	67			11		
N1	33.6	30.5	3.8	6.1	1.5	19.8
N2	42.9	48.1	2.6	4.5	1.9	30.5

Note: Percentage of SUs that exhibit significantly lower (first 3 columns) or higher (last 3 columns) FRs in the first of the 2 states indicated in the column header (RS vs. M, RSS vs. M, and RS vs. RSS) for all REST sessions (in 3 s slices).

The above findings are consistent in both monkeys, but the firing during sleepy rest (RSS) shows monkey specific differences. In monkey N, only 3–4% of all SUs show significantly lower FRs in RS than in RSS but in monkey E the percentage is $\approx $20% (Table 3 column 4). Vice versa, only 3.8% of all SUs in monkey E show significantly higher FRs in RS than in RSS, whereas this is true for $\approx $20% to 30% of all SUs in monkey N (last column). Similarly, the percentages of SUs with lower FRs during RS and RSS as compared with M (second and third column in Table 3) are similar in monkey N. In monkey E, however, only 25% of SUs show higher FRs during M than during RSS, whereas 38% of the SUs show a higher FRs during M as compared with RS. Thus, in agreement with our observations of the LFP spectra, rest and sleepy rest in monkey E express rather different features while they are quite similar to each other for monkey N.

Above we show that the firing of approximately half of the SUs is significantly correlated to the behavior and that RS is, on average, associated with lower FRs than movements. However, the absolute value of the FR alone is not predictive of the response of a SU to different behavioral states. In the following section, we aim to investigate other aspects than mere SU firing rate in different behavioral states.

### Single Unit Firing Properties in Spontaneous and Task-Related Behaviors

Given the relation between behavior and SU firing rate modulations, we now ask if other features of SU activity can be directly linked to particular behavioral states. From now on, we include R2G data to additionally look for differences on the level of spontaneous versus task-related behaviors.

**Figure 6:**
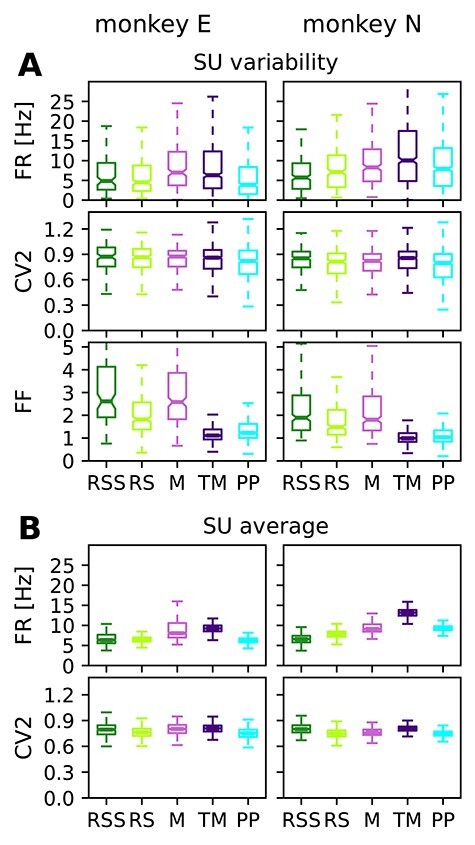
Comparison of firing properties in REST & R2G states calculated for 0.5 s data slices. (*A*) Box plots showing the variability across SUs: firing rate, spiking regularity, and spike count variability characterized by FR, CV2, and FF, here averaged over time slices. (*B*) Box plots showing the variability over time: distributions of time-resolved FR and CV2 averaged over SUs. Data pooled over REST sessions, two for each monkey (states RSS, RS, and M), and over R2G sessions, 6 of monkey E and 5 of monkey N (states TM and PP).

The box plots in Figure 6 and the values listed in Table 4 describe averaged FR, CV2 and the FF, calculated for 0.5 s time slices of all REST and R2G sessions, per SU and time slice (see Materials and Methods: Data Analysis). The CV2 characterizes the (ir-)regularity of neuronal firing across time. A value closer to zero (CV2 $\lessapprox $ 0.5) indicates regular spiking, the Poissonian firing is characterized by CV2 = 1 (Shinomoto *et al.* 2003; Voges and Perrinet 2010), and values higher than one indicate even more irregular spiking (characteristic, e.g., for certain types of gamma point processes, see Nawrot *et al.*  2008). The FF describes the variability of SU spike counts across trials (R2G) or time slices (REST) (Nawrot 2010; Nawrot *et al.* 2008; Riehle *et al.* 2018). It equals one for a Poisson process and decreases for more reliable spiking.

**Table 4:** Quantification of average FR (top row), regularity of spiking (CV2, middle row) and spike count variability (FF, bottom row)

Monkey	RSS	RS	M	TM	PP
	Firing rate FR [Hz]				
E	$6.67\pm 5.38$	$6.50\pm 5.74$	$8.86\pm 6.59$	$9.21\pm 9.29$	$6.30\pm 6.91$
N	$6.66\pm 4.67$	$7.83\pm 5.52$	$9.47\pm 6.32$	$13.26\pm 11.47$	$9.28\pm 7.06$
	Local coefficient of variation CV2				
E	$0.86\pm 0.16$	$0.83\pm 0.17$	$0.83\pm 0.15$	$0.85\pm 0.22$	$0.81\pm 0.24$
N	$0.83\pm 0.15$	$0.79\pm 0.15$	$0.80\pm 0.16$	$0.83\pm 0.17$	$0.78\pm 0.20$
	Fano factor FF				
E	$3.14\pm 1.55$	$2.06\pm 0.88$	$3.05\pm 1.72$	$1.30\pm 0.80$	$1.40\pm 0.63$
N	$2.31\pm 1.30$	$1.86\pm 1.07$	$2.29\pm 1.42$	$1.11\pm 0.59$	$1.20\pm 0.75$

Note: Given are mean values (averaged across time slices and SUs) and corresponding standard deviations with respect to SUs. All values are obtained from 0.5 s slices, for different behavioral states in REST (RSS, RS, M) and R2G (TM, PP), pooled across all recordings of the respective type.

Kruskal–Wallis tests for differences among the 5 behavioral conditions yield highly significant results for FR and FF ($P\ll 0.0001$) for both monkeys. The results of pairwise comparisons are listed in Table 5. Averaged across time slices (Fig. 6*A*), FRs are highest during movement states (M & TM) and lowest during rest RS(S) and PP (many differences being significant). Inferred from CV2, the firing is most regular in PP and least regular in RSS and TM, with a slightly larger spread of values in both R2G states as compared with REST, though these differences are mostly insignificant. In contrast, the differences in spike count variability as measured by FF are pronounced and statistically significant: R2G states exhibit a much smaller and less variable FF, that is, a higher reliability of spike counts across data slices, than REST states. Among the REST data, M and RSS show the highest mean and spread of FF values, whereas the spike count variability during RS is lower and less variable.

**Table 5:** Significance of pairwise comparisons of FR, CV2, and FF results shown in Figure 6*A*

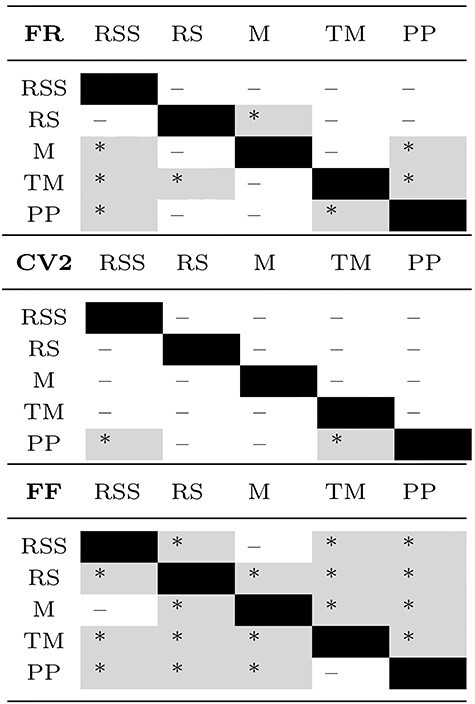

Note: Upper triangle of each table: monkey E; lower: monkey N. Stars indicate significant differences (*$P<0.001$) and minuses insignificant differences after Bonferroni–Holm correction with $\alpha =0.001$. Gray background highlights significant results.

Averaging across SUs (Fig. 6*B*), we also examine the variability in time. Note that even though the number of RS time slices highly exceeds that of SUs (cf. Table 1), the observed spread of the corresponding values is much smaller. This holds for all behavioral states. Since the variability across time slices (panel B) is much smaller than the variability across SUs (panel A), we later on average over time and consider only the variability with respect to SUs.

In summary, we find high SU variability in most of the measures for most of the states and the observed differences between states are mostly significant. Resting periods are characterized by rather low FRs as compared with movements in agreement with the results in section Results: Relation between Neuronal Firing  Rates and Behavior. The RS in particular shows a higher reliability of spike counts (lower FF) than M and RSS, but all REST states show a clearly higher FF as compared with R2G states.

### Network Firing Properties

**Figure 7:**
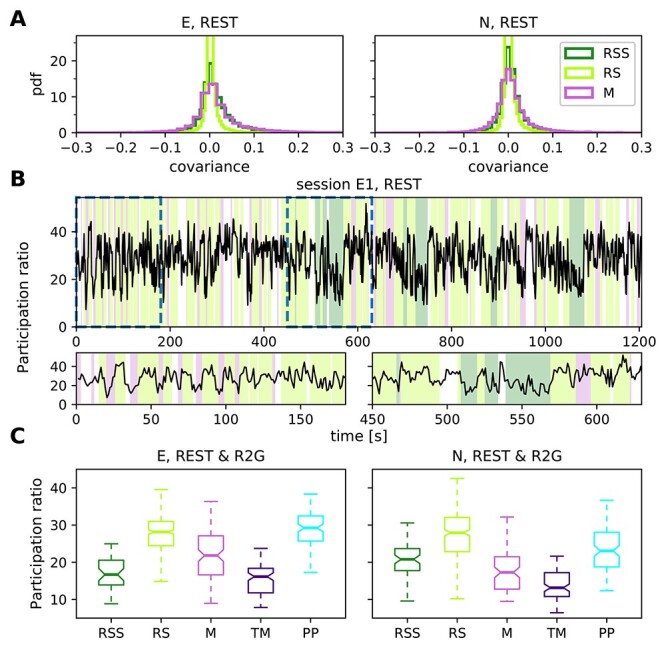
Network firing properties. (*A*) Distributions of pairwise COVs for the REST recordings of monkey E (left) and monkey N (right), calculated in 3 s slices with 100 ms bins, averaged over slices per SU pair and pooled over sessions. (*B*) Time-resolved PR in session E1, calculated in 3 s long sliding windows with an overlap of 2 s. Each value on the plot corresponds to the center of the respective window. Colors in the background indicate behavioral states (cf. legend in the right panel of A). Two bottom panels show close-up view at periods marked by dashed lines in the top panel. (*C*) Dimensionality: Box plots show the PR of REST (RSS, RS, M) and R2G (TM, PP) states for monkey E (left) and monkey N (right), each single value of the distributions corresponds to a single 3 s data slice. Pooled over sessions.

We now turn toward the analysis of coordinated firing as opposed to SU dynamics. Coordination between neurons can be measured at various time scales and quantified with various methods. We here consider spike count covariances calculated for 3 s slices with a bin size of 100 ms, see Materials and Methods:  Covariance and Dimensionality. To this end, we first show the COV distributions, averaged over slices of the REST data (Fig. 7*A*). Although the mean COV value during all behavioral states in REST is close to zero, the spread of the COV distribution differs between states. Statistical comparisons of the shapes of the distributions with 2-sample Kolmogorov–Smirnov tests reveal significant ($P\ll 0.0001$) differences for all pairs in both monkeys. In monkey E, the standard deviation of the COVs is considerably lower during RS ($\overline{\textrm{COV}}_{\textrm{RS}}=0.003\pm \mathbf{0.018}$) than during RSS ($\overline{\textrm{COV}}_{\textrm{RSS}}=0.018\pm \mathbf{0.058}$) and M ($\overline{\textrm{COV}}_{\textrm{M}}=0.02\pm \mathbf{0.08}$). The same is true for monkey N: $\overline{\textrm{COV}}_{\textrm{RS}}=0.005\pm \mathbf{0.021}$ compared with $\overline{\textrm{COV}}_{\textrm{RSS}}=0.01\pm \mathbf{0.042}$ and $\overline{\textrm{COV}}_{\textrm{M}}=0.01\pm \mathbf{0.055}$. For both monkeys, the mean COV value is the smallest during RS. In summary, we find that neuronal firing is much less correlated during rest as compared with movements.

The differences in the COV distributions motivate a more detailed investigation of the coordination of all recorded neurons. Apart from mean and variance of the COV distribution, another summarizing measure for the COV structure has been established and discussed in recent years: the participation ratio (Abbott *et al.* 2011; Mazzucato *et al.* 2016; Gao *et al.* 2017). The PR depends on all COVs in the network as it is derived from the eigenvalues of the COV matrix. Mazzucato *et al.*  (2016) show that the PR depends on a combination of the first and second order moments of auto- and cross-covariances. The physical interpretation of the PR is the dimensionality of the manifold spanned by the neuronal activity: a small PR indicates that only a few eigenvalues are necessary to account for a major fraction of the variance in the data, and hence only a few dimensions corresponding to the respective eigenvectors are needed to describe the dynamics of the activity, for example, in case of a coherent increase in neuronal firing of a majority of SUs. The higher the PR, the more dimensions are needed to capture the variability of the activity. We performed an analysis for the REST and also for the R2G states (0.5 s slices were concatenated to 3 s slices).

Figure 7*B* shows the time-resolved visualization of PR, calculated in a sliding window of 3 s length and 2 s overlap for 1 REST session of monkey E. The PR varies over time and it typically changes coherently with behavioral state (Fig. 7*B*, bottom panels). This observation is consistent across recordings and monkeys, as summarized in Figure 7*C*. The box plots visualize the distributions of PRs obtained in all nonoverlapping 3 s time slices from all recording sessions of a given type (REST or R2G) per monkey. These results indicate that the dimensionality of population spiking activity is highest during RS and PP, and lowest during TM. The PR during the RSS state is much lower than the one during the RS state, closer to the value obtained during movements, especially for monkey E. The spread of the values is notably higher in REST than in R2G states, especially in monkey N.

Kruskal–Wallis tests for PR differences among all behavioral conditions yield highly significant results ($P\ll 0.0001$) for both monkeys. Pairwise comparisons yield mostly significant results except for RS versus PP and RSS versus TM in monkey E, as well as RSS versus M and PP in monkey N. These results hold for different bin sizes (see Materials and Methods: Covariance and  Dimensionality).

The higher dimensionality of RS and PP as compared with movement states and sleepy rest is a clear evidence for the complexity of these states: more dimensions are needed to capture their activity dynamics. Moreover, the large difference between the PR of RS and RSS emphasizes the necessity to distinguish between rest with eyes open and closed. In the following, we will support this claim by analyzing the balance between putative excitatory and inhibitory population activity.

### Balance in Population Activity

Complementary to the dimensionality analysis that is sensitive to single neuron-specific fluctuations, we now investigate the population-level coordination that depends only on average single-neuron COVs. Balance between excitation and inhibition is considered as an attribute of a physiological network state in contrast to nonphysiological states like, for example, epilepsy, though transient deviations from perfect balance, that is, an instantaneous dominance of excitation or inhibition, have been observed during physiological activity (Zhang and Sun 2011; Dehghani *et al.* 2016). Theoretical studies simulating cortical network dynamics mostly assume a balanced resting state (Vreeswijk and Sompolinsky 1996, 1998; Brunel 2000) and relate this to low average COVs between neurons (Renart *et al.* 2010; Tetzlaff *et al.* 2012). We here investigate the balance between putative excitatory (bs) and inhibitory (ns) population activities across different behavioral states.

To begin with, we compute FRs in 0.5 s slices averaged separately over bs and ns SUs across all REST and R2G sessions and states: The average FR of ns SUs is 9.93$ \pm $9.33 spikes/s for monkey E and 11.97 $\pm $ 9.48 spikes/s for monkey N, whereas the average FR of bs SUs is considerably lower, namely 5.73 $\pm $ 5.54 spikes/s for monkey E and 8.51 $\pm $ 7.49 spikes/s for monkey N. The same tendency can be observed when considering separate behavioral states in REST recordings (Table 6): Putative inhibitory neurons exhibit higher FRs than putative excitatory cells, and the ratio between the rates (ns/bs) is always larger than one. There is, however, no specific relation between the ns/bs ratio and the behavioral state.

**Table 6:** Average FRs of bs and ns SUs and their ratio

Session	RSS			RS			M		
	bs	ns	ns:bs	bs	ns	ns:bs	bs	ns	ns:bs
E1	$4.38\pm 3.11$	$8.68\pm 6.1$	2.0	$4.17\pm 3.36$	$8.43\pm 7.18$	2.0	$5.8\pm 5.14$	$10.67\pm 7.57$	1.8
E2				$4.94\pm 4.04$	$8.71\pm 6.69$	1.8	$6.91\pm 4.53$	$12.05\pm 7.14$	1.7
N1	$5.67\pm 4.5$	$8.22\pm 5.24$	1.4	$6.7\pm 5.51$	$9.24\pm 5.56$	1.4	$7.26\pm 5.33$	$12.19\pm 6.32$	1.7
N2	$5.92\pm 4.09$	$7.55\pm 4.28$	1.3	$6.77\pm 4.88$	$9.3\pm 5.57$	1.4	$7.72\pm 5.07$	$12.16\pm 7.27$	1.6

Note: FRs are computed from 3 s slices in 4 REST sessions and separately for each REST state (RSS, RS, and M).

**Figure 8:**
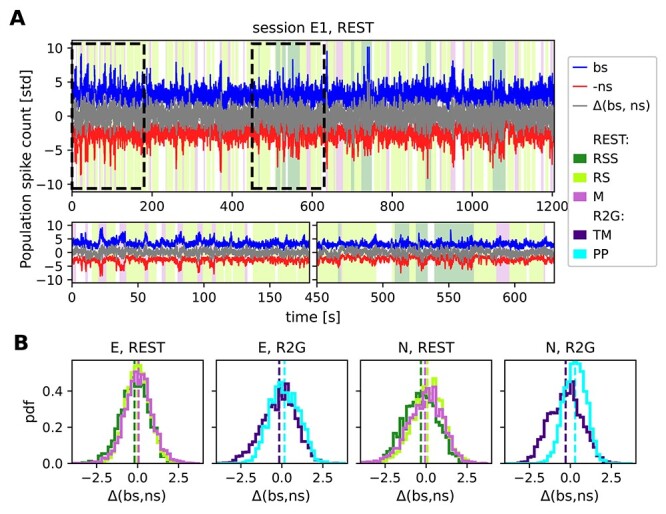
Balance between putative excitatory and inhibitory population activity. (*A*) Population activities and the difference (gray) between z-scored putative excitatory (bs, blue) and inhibitory (ns, red) firing during a single REST session of monkey E on a 100 ms time scale. Colors in the background denote behavioral states (cf. Fig. 4*A*). For a better visualization, spike counts of bs and ns populations are normalized by their standard deviation instead of z-scoring. Additionally, ns time series is multiplied by (−1). (*B*) Histograms of the difference between globally z-scored population activities of putative excitatory (bs) and inhibitory (ns) SUs of all REST (left) and R2G sessions (right) for monkey E (first 2 panels) and monkey N (last 2 panels), calculated in 100 ms bins. Results are pooled across all recordings of the respective type.

Therefore, we now look at a much smaller time scale and at the balance defined as the difference between ns and bs population spiking. Figure 8*A* presents time-resolved, z-scored spike counts of bs (blue) and ns (red) population in 100 ms bins, and the difference between the population activities in gray. Putative excitatory and inhibitory activities seem to fluctuate simultaneously, indicating balance: Pronounced deviations from average spike counts can be seen in both populations, especially during RSS (dark green background color) and M (pink background). Considering the distributions of mean population spike counts (Supplement 1  Fig. 1), the standard deviations during M and RSS (9.56 $\pm $ 2.49 and 7.81 $\pm $ 2.75, respectively, ns population, session E1) are much higher than during RS (7.56 $\pm $ 1.54). They are even larger (approximately factor 1.7) than expected from the larger means (approximately factor 1.1)—an indication of distributions with more extreme values, that is, potential transient increases in the population spike count.

We performed a quantitative analysis of how the balance between bs and ns SUs relates to the behavioral states, on the time scale of 100 ms, for our REST and R2G data. Firstly, we asked if there was a state-specific prevalence of ns or bs activity. Figure 8*B* shows the results of subtracting z-scored ns from z-scored bs population activity. The histograms show the distributions of values obtained in all 100 ms bins in the pooled REST and R2G sessions of monkey E and N, and Table 7 (top row) lists the values of mean and standard deviation.

**Table 7:** Quantification and correlation of balance and dimensionality

Monkey	RSS	RS	M	TM	PP
	Putative excitatory/inhibitory prevalence $\Delta $ (bs, ns)				
E	$-0.19\pm 0.86$	$0.00\pm 0.72$	$0.06\pm 0.85$	$-0.16\pm 1.04$	$0.16\pm 0.89$
N	$-0.32\pm 1.01$	$0.09\pm 0.98$	$-0.06\pm 1.07$	$-0.28\pm 0.97$	$0.28\pm 0.67$
	Instantaneous balance $\rho (\textrm{bs},\textrm{ns})$				
E	$0.39\pm 0.22$	$0.21\pm 0.23$	$0.44\pm 0.29$	$0.27\pm 0.22$	$0.1\pm 0.21$
N	$0.36\pm 0.21$	$0.16\pm 0.24$	$0.16\pm 0.24$	$0.39\pm 0.24$	$0.08\pm 0.21$
	Participation ratio (PR)				
E	$17.2\pm 4.4$	$27.5\pm 5.1$	$21.7\pm 6.9$	$15.4\pm 3.9$	$28.9\pm 4.9$
N	$21.0\pm 5.9$	$27.3\pm 6.8$	$18.0\pm 6.3$	$13.7\pm 4.1$	$23.4\pm 5.5$
	Spearman rank correlation between $\rho (\textrm{bs},\textrm{ns})$ and PR				
E	–0.28	–0.13 ($P<0.01$)	–0.63 ($P\ll 0.001$)	–0.15	–0.15
N	–0.19	–0.14 ($P<0.05$)	–0.29 ($P<0.01$)	–0.29 ($P<0.01$)	0.16

Note: Top rows: quantification of balance between putative excitatory and inhibitory population activities ($\Delta $(bs, ns) and $\rho (\textrm{bs},\textrm{ns})$). Middle row: quantification of dimensionality as measured by the PR. Bottom row: Spearman rank correlation between $\rho (\textrm{bs},\textrm{ns})$ and PR; only $P$ values smaller than 0.05 are listed. All values were obtained from 3 s slices, for different behavioral states in REST (RSS, RS, M) and R2G (TM, PP), pooled across all recordings of the respective type.

We find a clear difference between the distributions obtained for the PP and TM in the R2G data of both monkeys: TM distributions are shifted toward negative and PP toward positive difference values (mean $\pm $ std: $\overline{\Delta }_{\textrm{TM}}=-0.16\pm 1.04$, $\overline{\Delta }_{\textrm{PP}}=0.16\pm 0.89$ for monkey E and $\overline{\Delta }_{\textrm{TM}}=-0.28\pm 0.97$, $\overline{\Delta }_{\textrm{PP}}=0.28\pm 0.67$ for monkey N), pointing out a prevalence of ns or bs activity, respectively. This indicates that the balance between the excitatory and inhibitory activity dynamically changes depending on the behavioral state of the monkey during task performance.

In the REST data of both monkeys, the sleepy rest state is slightly dominated by inhibition ($\overline{\Delta }_{\textrm{RSS}}=-0.19\pm 0.86$ for monkey E and $\overline{\Delta }_{\textrm{RSS}}=-0.32\pm 1.01$ for monkey N). Concerning rest and movements, however, the general tendencies are less pronounced: For monkey E, we find $\overline{\Delta }_{\textrm{RS}}=0\pm 0.72$ and $\overline{\Delta }_{\textrm{M}}=0.06\pm 0.85$, thus no particular dominance. For monkey N, we find $\overline{\Delta }_{\textrm{RS}}=0.09\pm 0.98$ and $\overline{\Delta }_{\textrm{M}}=-0.06\pm 1.07$, so again not significantly dominated by any population.

**Figure 9:**
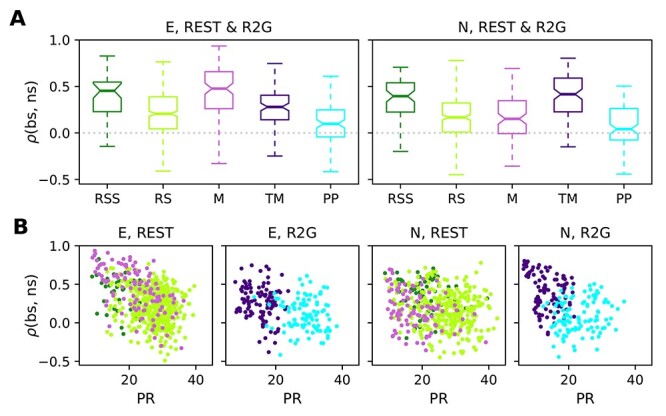
Instantaneous balance and its relation to dimensionality. (*A*) Box plots of the correlation between putative excitatory and inhibitory population activity calculated in 3 s slices, which quantifies the instantaneous (100 ms) balance for monkey E (left) and N (right). (*B*) Scatter plots showing the relationship between the instantaneous balance $\rho (\textrm{bs},\textrm{ns})$ and dimensionality PR, for monkey E (panels on the left) and N (panels on the right). Each dot represents the PR and $\rho (\textrm{bs},\textrm{ns})$ values of one 3 s slice during REST or R2G recording. Results are pooled across all recordings of the respective type. Color code is the same as in Figure 7.

Secondly, in addition to the mere difference between the bs and ns activities, we quantified how they covary with each other within each time slice by computing the Spearman rank correlation $\rho (\textrm{bs},\textrm{ns})$ between bs and ns population activity (cf. Renart *et al.*  2010; Tetzlaff *et al.*  2012). A higher correlation value indicates a more strict instantaneous balancing between the excitatory and inhibitory activity. Figure 9*A* shows box plots of the correlation measure $\rho (\textrm{bs},\textrm{ns})$ for the different behavioral states of the 2 monkeys, and the corresponding means and standard deviations are listed in Table 7. For monkey E, the correlation between bs and ns activity is highest during M ($\overline{\rho }_{\textrm{M}}=0.44\pm 0.29$), meaning that the balance was kept best during M state, closely followed by RSS ($\overline{\rho }_{\textrm{RSS}}=0.39\pm 0.22$), see Figure 9A, left. RS shows the lowest correlation ($\overline{\rho }_{\textrm{RS}}=0.21\pm 0.23$), it is thus the least balanced state in the REST recordings. Pairwise comparisons confirm significantly different results for RS versus RSS and M, but not for RSS versus M. In monkey N, RS and M exhibit identical mean correlations ($\overline{\rho }_{\textrm{RS}}=0.16\pm 0.24$, $\overline{\rho }_{\textrm{M}}=0.16\pm 0.24$) (see Fig. 9*A*, right), both are significantly less balanced than RSS ($\overline{\rho }_{\textrm{RSS}}=0.36\pm 0.21$).

In the R2G data of both monkeys, we find that the PP ($\overline{\rho }_{\textrm{PP}}=0.1\pm 0.21$ for monkey E and $\overline{\rho }_{\textrm{PP}}=0.08\pm 0.21$ for monkey N) is less balanced than task-induced movements ($\overline{\rho }_{\textrm{TM}}=0.27\pm 0.22$ for monkey E and $\overline{\rho }_{\textrm{TM}}=0.39\pm 0.24$ for monkey N); the PP state shows a significantly ($P<0.001$) lower correlation between ns and bs activities. We thus conclude that behavioral states without movements (RS, PP) are less balanced than movement states when considering a timescale of 100 ms.

Participation ratio and balance measure different aspects of correlations in the underlying network. We now ask if and how these measures relate to each other. To this end, we analyzed the relation of PR and $\rho (\textrm{bs},\textrm{ns})$ using scatter plots (Fig. 9*B*)—each 3s slice is represented by a single data point. The points are colored according to the behavioral state they are computed from. For the REST data, we observe a negative correlation between PR and $\rho (\textrm{bs},\textrm{ns})$ (see Table 7): The higher the complexity, the lower the balance. Data points from different behavioral states overlap strongly and are thus not clearly separable. In contrast, TM and PP of the R2G data separate into 2 different clouds according to their PR, but there is no clear correlation to $\rho (\textrm{bs},\textrm{ns})$.

## Discussion

Experiments without any imposed stimuli or task have been investigated in numerous studies and referred to with multiple names: 1) ongoing, intrinsic, or baseline activity of single brain areas (Arieli *et al.* 1996; Tsodyks *et al.* 1999) in anesthetized animals, 2) spontaneous or resting state activity on the whole brain level (Vincent *et al.* 2007; Raichle 2009; Deco *et al.* 2011), and 3) idle state of point-neuron network simulations (Brunel 2000; Potjans and Diesmann 2014; Dahmen *et al.* 2019). Yet, a thorough characterization of spiking activity in the awake resting condition on the level of single neurons was still missing.

Here, we investigate the properties of spiking activity in macaque motor cortex during 5 behavioral states: resting state (no movements, RS), sleepy rest (no movements with eyes closed, RSS), spontaneous movement (M), task-related movement (TM) and task-imposed waiting without movements (PP), with a particular focus on RS. Our main findings are: 1) we demonstrate a considerable correlation between neuronal firing and behavior, 2) we find that RS SU activity is characterized by relatively low average firing rates and a high variability of spike counts across data slices, 3) compared with other states, we identify a higher dimensionality of the joint activity during RS, which is 4) correlated with a low level of balance between putative excitatory and inhibitory population spiking.

### Single Unit Activity and LFP during Different Behaviors

Many studies investigate the link between neuronal activity in the motor cortex and behavior using LFP data (e.g., Pfurtscheller and Aranibar  1979; Fontanini and Katz 2008; Engel and Fries 2010; Kilavik *et al.* 2013). Low-frequency oscillations (<15 Hz) are often linked to sleep (Gervasoni *et al.* 2004; Fontanini and Katz 2008), beta oscillations ($\approx $13-30 Hz) typically appear during movement preparation or postural maintenance (Baker *et al.* 1999; Kilavik *et al.* 2012), whereas faster oscillations mostly reflect attention and neuronal processing during movements (Liu and Newsome 2006; Fontanini and Katz 2008). Our visual classification of the behavior is in good agreement with the LFP characteristics shown in the above studies.

Firstly, all states without movements (RSS, RS, and PP) show pronounced beta oscillations that are shifted toward higher frequencies during task-imposed rest (PP) compared with spontaneous rest (RS). Secondly, both spontaneous and task-related movements (M and TM) show stronger fast oscillations than nonmovement states. The spectra obtained during RSS (eyes closed) indicate distinct physiological states in the 2 monkeys: the peak frequency during RSS of monkey E occurs at a much lower frequency compared with monkey N. This suggests that closing the eyes indicates drowsiness in monkey E but not necessarily in monkey N.

Furthermore, in agreement with previous studies on behaving monkeys (Nawrot *et al.* 2008; Rickert *et al.* 2009; Churchland *et al.* 2010; Nawrot 2010; Riehle *et al.* 2018), we find that the spiking activity is highly variable across SUs and that the average FR is increased during movements as compared with waiting for the cue at rest. In REST data, we find a significant correlation between SU firing and the monkey’s behavior. This indicates that the analysis of spiking activity is another valid approach next to LFP and large-scale recordings to investigate behavioral states, including resting state. Analogously to activations and deactivations of specific brain areas reported in fMRI studies (Biswal *et al.* 1995; Raichle 2009; Deco *et al.* 2011), we observe systematic increases and decreases in firing rates in numerous SUs. Also in agreement with Nawrot *et al.*  (2008), Rickert *et al.*  (2009), Churchland *et al.*  (2010), Nawrot  (2010), Riehle *et al.*  (2018), we find a lower spike count variability during task-related movements (TM) than during movement preparation (PP) and vice versa for the spike time irregularity (Our results are less significant than those presented in Riehle *et al.*  (2018); we analyze only a subset of the R2G data and use partially different methods.).

A new finding of our study is a pronounced difference in variability between REST and R2G states, that is, between spontaneous and task-related behavior. All REST states show a significantly higher spike count variability than the R2G states. These differences are probably due to the behavioral constraints present in the R2G but not in the REST experiments. During R2G tasks, the monkey received visual input to control periods of waiting or arm movements, resulting in well-defined behavioral states and partially constrained mental states with a more regular and reliable firing. In contrast, during REST experiments, the monkey itself decided what to do (e.g., movement preparation or onset), resulting in a less well-defined behavior and its timing.

The above findings are consistent for the 2 monkeys, but there are differences concerning the sleepy resting state: For monkey E, FRs during RSS are higher than during RS, thus closer to the values measured during M, whereas this is not the case for monkey N. Thus, in agreement with what we find for the LFP spectra, the distinction between RS and RSS (eyes open versus eyes closed) is more pronounced in monkey E than in monkey N.

### Network Activity

During all behavioral states, the network activity of groups of neurons in the motor cortex is characterized by a dimensionality much lower than the maximal possible dimension, that is, the total number of recorded single neurons. Task-related movements show the lowest dimensionality, expressed by a small PR, whereas nonmovement states show a significantly higher dimensionality. Accordingly, neuronal firing during rest is less coordinated than during other states, as indicated by the narrower COV distribution centered at zero. These findings agree well with Mazzucato *et al.*  (2016), Gao *et al.*  (2017) who compare stimulus-evoked and ongoing neuronal activity, assuming M and TM to represent the evoked activity, and RS and PP the ongoing activity. The low mean PR of approximately 13–15 (compared with the possible maximum value of 89 given by the number of recorded units) during TM (Fig. 7) shows that the neural state space dynamics of the R2G movement can be reconstructed from only a few principal components. In contrast, the ongoing activity during RS and PP is of significantly higher dimensionality ($\approx $27 and $\approx $23–29, respectively) and thus more complex.

In accordance with Csicsvari *et al.*  (1999), Peyrache *et al.*  (2012), Dehghani *et al.*  (2016), we also find that putative excitatory and inhibitory population spiking are primarily well balanced. However, our detailed time-resolved analysis, that is, calculating the balance in 100 ms bins, uncovers the following particularities. During R2G experiments, the activity alternates between excitation-dominated movement preparation (PP) and inhibition-dominated movement execution (TM). During nonmovement states (PP and RS), we find a reduced correlation between putative excitation and inhibition, that is, a reduced instantaneous balance of nonmovement states. In addition, the instantaneous balance is anticorrelated to the dimensionality, particularly strongly in REST.

We suspect that the relatively high instantaneous balance during movements and sleepy rest is partially an effect of an enhanced number of transient changes in population spiking in these states as compared with the other states (Fig. 8*A*). A prominent increase in firing as observed during movements is an unambiguous type of activity change and is thus easy to capture by correlation measures. Such transient increases correlated in time between 2 neuronal populations could result from the recurrent coupling between excitatory and inhibitory neurons (see Supplement 1). In addition to the transients in population activity, we find hints of a prevalence of nonstationarities (e.g., transients) in the SU firing during movements and sleepiness, but not during rest (Supplement 1  Fig. 2) Strong transient comodulations of spiking activities amplify correlations between neurons, which in turn decrease the dimensionality of network activity. Therefore, transient changes in FRs might also be partially responsible for the reduced dimensionality during task-related movements.

### Influence of Preprocessing and Critical Assumptions

Specificities of extracellular recordings and the following preprocessing steps impose particular biases on the resulting statistics and their interpretation. Firstly, the spike sorting procedure, necessary to identify single cells recorded on the same electrode, is well-known to be problematic (Lewicki 1998; Quian Quiroga 2012). Additional limitations on minimal SNR and FR of a sorted unit to be considered for statistical evaluation contributes to the undersampling of sparsely firing neurons and thus biases results toward highly active neurons. This is often referred to as the problem of “dark matter” of the brain (Shoham *et al.* 2006).

Secondly, the separation between putative excitatory and inhibitory neurons based on the widths of their spike waveforms is known to have several limitations but it is still a widely-used approach (Bartho *et al.* 2004; Kaufman *et al.* 2010, 2013; Peyrache *et al.* 2012; Dehghani *et al.* 2016; Peyrache and Destexhe 2019). Some pyramidal neurons, in particular when recorded close to the axon, exhibit narrow waveforms (Vigneswaran *et al.* 2011) and approximately 10% of M1 interneurons have intermediate or broad waveforms (Merchant *et al.* 2008; Kaufman *et al.* 2013). Thus, when discussing the differences between the 2 populations, it should be kept in mind that not all ns units are inhibitory and only the majority of bs SUs are excitatory (Peyrache and Destexhe 2019). Nevertheless, our separation yields consistently higher average FRs for putative inhibitory neurons, both when considering single behavioral states in every REST session (see Table 6) and when looking at the mean across all behaviors (REST and R2G), which agrees well with what is known from the literature (Kaufman *et al.* 2010; Peyrache *et al.* 2012; Dehghani *et al.* 2016). The average difference is as high as $\approx $3 spikes/s for monkey N and $\approx $4 spikes/s for monkey E: a 1.5–2-fold difference in FR between the ns and bs population.

Thirdly, our study relies on the behavioral segmentation of REST recordings, which is highly subjective and has rather poor temporal resolution (${\sim }$1 s) in comparison to the recorded neuronal activity (${\sim }$1 ms). Nevertheless, our behavioral classification seems to be accurate in terms of separating sets of dissimilar neurophysiological network states, as reflected by differences in state-resolved LFP spectra, see above. Still, our definitions of the behavioral states are based on visual inspection and may not be as precise. For example, the identification of “whole body and limb movements” in the video recording does not account for the fact that, due to the exact placement of the Utah array, our recordings are particularly sensitive to contra-lateral arm movements. Likewise, the RS classification is simply based on the exclusion of movements with the additional criterion of “eyes open.” Compared with the very precise behavioral classification in R2G recordings—for example, PP is defined as 500 ms after CUE-OFF when the monkey is forbidden to move, and constraining the analyzed data to only successful trials ensures that the monkey was focused on the upcoming cue to perform the task—the behavioral segmentation of REST recordings is vague and allows for a much broader range of actual behaviors.

Finally, reliable covariance estimation necessitates very long data slices (Cohen and Kohn 2011). To satisfy this requirement, in R2G data, we had to concatenate slices from 6 consecutive trials into 3 s slices for the analysis of COV and PR. Thus, a single PR value results from averaging over 6 independent recording periods in contrast to the continuous REST data. However, this approach can be justified by our observation of a low inter-trial variability obtained for 0.5 s slices of the R2G data.

### Toward Experimental Data for Spiking Model Validation

Modeling studies focusing on spiking-neuron networks often claim to model an idle state, that is, without any relation to functional aspects, characterized by sparse asynchronous irregular spiking and balanced input statistics (Amit and Brunel 1997; Vreeswijk and Sompolinsky 1996, 1998; Brunel 2000; Kumar *et al.* 2008; Voges and Perrinet 2010, 2012; Potjans and Diesmann 2014). To isolate the ongoing and recurrently generated activity, many of these studies consider stationary states without any transient network activations due to external impacts (e.g., stimuli or drugs). In this case, single-neuron and population FRs fluctuate around some mean activity. However, data collected in behavioral experiments often contain transient FR fluctuations on the level of both SUs and whole populations. For motor cortex recordings, such FR changes typically occur during movements, which has been shown here and in many other studies (Nawrot *et al.* 2008; Rickert *et al.* 2009; Churchland *et al.* 2010; Riehle *et al.* 2018, 2013). We find that this disagreement can (mostly) be avoided by considering nonsleepy resting periods (RS) in REST recordings only. Using nonmovement epochs (PP) during behavioral tasks yields results that are more similar to RS in terms of network firing properties, but the SU variability is still different (much lower for FF). A comparison to inappropriate data sets could lead to erroneous conclusions on model parameters and on the mechanisms that shape the network dynamics. Hence, network models that claim to mimic an idle state in terms of SU and network activity should ideally be validated against awake resting state data.

#### Balance and Correlations

The modeling literature discusses different types of balance (Deneve and Machens 2016), which generally refers to a cancelation of excitation and inhibition in the neuronal input. One distinguishes between a static and a dynamic view on balance. The static view focuses on the strength and number of excitatory and inhibitory afferent connections (Poil *et al.* 2012), leading to dynamics characterized by avalanche-like behavior (Beggs and Plenz 2003). Dynamical balance, also called the balanced state (Amit and Brunel 1997; Vreeswijk and Sompolinsky 1996, 1998; Brunel 2000), emerges when excitatory and inhibitory inputs cancel each other at each point in time (Renart *et al.* 2010) as an effect of excess inhibitory feedback (Tetzlaff *et al.* 2012). Our extracellular recordings do not allow us to assess inputs to neurons, we therefore study balance based on neuronal outputs in form of correlations between population activities, similar to Dehghani *et al.*  (2016). Input and output correlations between excitatory and inhibitory activities are related in recurrent networks; their mapping is, however, not unique (Helias *et al.* 2014): Often a cancelation of inputs to a single neuron is associated with strong correlations between excitatory and inhibitory spiking of the network (Renart *et al.* 2010). However, balanced networks can also exhibit deviations between population activities if the latter are organized such that their net effects on the summed input to single neurons cancel out (Tetzlaff *et al.* 2012; Baker *et al.* 2019). The Balance of population activities is therefore not fully informative on the amount of balance in inputs. We here find principally well-balanced population firing in all behavioral states, and we show that spiking during rest with eyes open is neither dominated by excitation nor by inhibition. The apparent reduction of instantaneous balance in RS periods could be an effect of fewer transient activities contributing to the correlation between excitatory and inhibitory population firing (cf. Supplement 1). As discussed above, this is still consistent with predictions from balanced network models. Our results on balance in RS and the differences from periods with activity transients can therefore be used to constrain spontaneous and evoked population activities in network models.

Related to balance, modelers often assume uncorrelated or weakly correlated external inputs to local networks, but it is impossible to determine the amount of correlations in the neuronal input with extracellular recordings. Strongly correlated inputs, attributed to sensory (Decharms and Merzenich 1996) or movement processing (Murphy *et al.* 1985), may boost the modulation of FRs on the population level. This could lead to higher pairwise COVs and subsequently lower dimensionalities than expected in artificial networks with a well-controlled input structure. We find that such a decrease in dimensionality is, for example, particularly pronounced during task-induced movements. This again points out the necessity to separate between rest and movements in order to avoid potential unrealistic mismatch between input and output statistics of spiking models.

#### Heterogeneity of Neuronal Networks

Another point is the remarkable heterogeneity of neuronal activities in experimental recordings: SUs show a broad range of FR profiles and spiking (ir-)regularities, as well as distinct activity modulation related to behavioral state changes. Neuronal network studies mostly are able to reproduce this heterogeneity. Single-neuron properties (e.g., time constants, synaptic weights) and connectivities are typically given as parameter ranges described by certain distributions derived from experimental measurements (Kumar *et al.* 2008; Voges and Perrinet 2012; Potjans and Diesmann 2014). Depending on the widths of these distributions (and other features), the resulting activities can and should be adapted to the heterogeneity in experimental data (Dahmen *et al.* 2019). An advantage of heterogeneous network activity is that it enhances the stability of the idle state (Denker *et al.* 2004), which is essential for real-world neuronal networks that need to be able to operate under various conditions. For example, the different behavioral states analyzed here demonstrate that the motor cortex operates in similar dynamical regimes for various kinds of behaviors, including movements and sleepiness. The stability range of network models can be further increased by including more real-world features like homeostatic mechanisms (e.g., adaptation, short-term plasticity) which also support a high (temporal) heterogeneity.

In summary, we encourage modelers to (continue to) incorporate the heterogeneity of real-world neuronal activities, and we conclude that the validation of network models that claim to simulate idle states should be based on resting state data from awake subjects. Still, even when considering REST recordings without any task or stimulus, it is necessary to separate out the “pure” resting state periods with eyes open because they show distinct statistical properties: lower FRs, fewer transient activities, smaller COVs and thus a higher dimensionality.

### Definition of Behavioral States

The rather vague classification of behavioral states in REST recordings is based on observing the monkey in contrast to the precise classification in R2G experiments, which relies on external cues. The consequence of this difference in precision is clearly visible on the level of the spiking activity statistics: In addition to the higher (broadly distributed) spike count variability in REST compared with R2G, REST states also show a less clear state-specific difference in the dimensionality results.

In addition, there is the problem of different time scales (i.e., slice lengths): 0.5 s as forced by the R2G settings versus the heuristically chosen 3 s in REST. Thus, some comparisons between single behavioral states of these 2 data types might be unfair, but we still observe the expected commonalities in the states with (TM, M) versus without movements (PP, RS): Nonmovement states show generally lower FRs, a higher dimensionality, and a lower instantaneous balance.

#### Eyes Open versus Closed

The sleepy resting state RSS turns out to be a special case. As already mentioned, the LFP spectra and the firing statistics of RSS are monkey-specific: in monkey E, the distinction between RS and RSS is more pronounced than in monkey N. RS and RSS can be distinct physiological states in a given monkey: monkey E seems to be really drowsy when its eyes are closed, whereas monkey N might be simply bored. It is known that the neuronal activities in awake subjects show considerable differences from sleep (Gervasoni *et al.* 2004) or anesthesia (Nelson *et al.* 2004). This example shows the importance of verifying the result of the visual behavioral segmentation with the LFP spectra of the resulting states.

However, concerning both dimensionality and instantaneous balance, the RSS distributions of the monkeys are similar. In addition, in both monkeys mean dimensionalities are closer to the ones obtained for M than for RS, even though RSS is a nonmoving state. In accordance with observations that the motor cortex can show distinct reactions to visual stimuli (Wannier *et al.* 1989; Riehle 1991), we conclude that the distinction between eyes open and eyes closed is indispensable even in the motor cortex, since there is an impact on the neuronal activity.

#### Alternative Classification Methods

There are other possibilities for the behavioral segmentation of REST recordings. One idea would be an automatic decoding of behavioral state purely based on SU firing properties by means of machine learning methods, for example shown in Pandarinath *et al.*  (2018). Given that approximately 50% of all SUs exhibit a strong correlation between FR modulations and behavior, such an approach would probably be possible but not necessarily straightforward. If there were enough data to define an appropriate learn set, a machine learning algorithm could, for example, identify SUs that consistently increase or decrease their FR with specific state changes. Such an approach, however, is beyond the scope of this study. Another idea would be to increase the temporal precision of the visual segmentation by means of an automated detection of transient neuronal activities. Yet, the detection of transient activities in itself is not trivial (Ito *et al.* 2019), it does not allow to distinguish between RSS and M, and particularly in our data a 3 s long movement epoch contains several such transients in an unknown frequency. We do not pursue this approach, as it is again beyond the scope of this study.

#### Resting State as Superposition of Subnetwork Activities

An interesting hypothesis emerges from the comparison of our study to resting state studies based on large-scale measurements. Similar to the observation of activations and deactivations of specific brain areas in fMRI studies (Biswal *et al.* 1995; Fox and Raichle 2007; Raichle 2009; Deco *et al.* 2011; Heuvel and Hulshoff Pol 2010), we observe systematic in- and decreases in the spiking activity of numerous SUs. Large-scale studies conclude that spontaneous brain activity emerges from a set of resting state networks (RSNs) (Fox and Raichle 2007; Raichle 2009; Heuvel and Hulshoff Pol 2010; Deco *et al.* 2011), that is, from a sequence of consistently re-occurring spatio-temporal activity patterns that resemble task-evoked activity, but are present during rest (Fox and Raichle 2007; Vincent *et al.* 2007; Heuvel and Hulshoff Pol 2010). One could thus hypothesize a similar phenomenon on the microscopic level of spiking activity: a resting state composed of the activities of several subnetworks of single neurons in the motor cortex. During movements, one could imagine a convergence of the neuronal activity into specific networks (cf. Fox and Raichle 2007; Mazzucato *et al.* 2016). The larger spatial spread of the activity observed during RS compared with M (see Supplement 2) would be in line with the above hypothesis, assuming that a superposition of many spatially embedded networks yields an enlarged spatial extent as compared with a single such network (cf. Fig. 1 in Supplement 2). Likewise, the high dimensionality observed during RS agrees well with the hypothesis of a superposition of several subnetworks.

Yet another question concerns the definition of “rest” in general: how to define it in other cortical areas than motor cortex, for example, in sensory systems? For the auditory system one would intuitively assume that silence or white noise as auditory input represents the resting condition. Similarly, for the visual system one could use a uniform or noise background as visual input. The choice of ”eyes-closed” as rest condition would, however, represent a different behavioral state compared with our assumption of sleepy rest being a qualitatively different condition.

Given all the issues concerning the definition of “rest” and the behavioral segmentation, together with the superposition of RSNs on the scale of brain areas, one could claim that it is futile to attempt to characterize the spiking activity during an assumed resting state. However, our results clearly demonstrate a set of significant differences between the spiking activity in motor cortex during “rest” as compared with other behavioral conditions.

## Conclusion

We demonstrate that spiking activity in monkey motor cortex during rest differs significantly from other spontaneous and task-related behavioral states, for example sleepiness and movements. The main characteristics of the resting state activity are low average firing rates combined with a high variability of SU spiking statistics, and a pronounced complexity as indicated by a less coordinated firing, which results in a higher dimensionality of the network activity. We show that and explain why neuronal network models should be validated against resting state data, aiming to enhance the trend toward realistic network models that account for the heterogeneity in neuronal data. We hope that our study is just the beginning of the characterization of “rest” on the level of spiking neurons. More specific analysis is needed to quantify transient activities, their relation to the balance between excitatory and inhibitory population activities, and to provide an automated algorithm for the behavioral segmentation of REST recordings.

## Funding

Deutsche Forschungsgemeinschaft (GR 1753/4-2, DE 2175/2-1) Priority Program (SPP 1665); the Helmholtz Association through the Helmholtz Portfolio Theme Supercomputing and Modeling for the Human Brain; the European Union’s Horizon 2020 Framework Programme for Research and Innovation (No. 720270, 785907) (Human Brain Project SGA1 and SGA2) and Agence Nationale de la Recherche (ANR-11-BSV4-0026 “GRASP”), Collaborative Research Agreements RIKEN-CNRS, and FZ Juelich-CNRS.

## Supplementary Material

Resting_state_dynamics_S1_REVIEWED_ACCEPTED_tgab033

Resting_state_dynamics_S2_REVIEWED_ACCEPTED_tgab033

Resting_state_dynamics_S3_REVIEWED_ACCEPTED_tgab033

Resting_state_dynamics_S4_REVIEWED_ACCEPTED_tgab033

## Notes

We thank Tom Tetzlaff and Alper Yegenoglu for fruitful discussions.
