# Supplementary material for: On the Complexity of Resting State Spiking Activity in Monkey Motor Cortex
Source: Cereb Cortex Commun. 2021 May 18;2(3):tgab033. doi: 10.1093/texcom/tgab033 (PMC8271144; doi:10.1093/texcom/tgab033)
Supplement: Resting_state_dynamics_S1_REVIEWED_ACCEPTED_tgab033 [file resting_state_dynamics_s1_reviewed_accepted_tgab033.pdf]

Supplement 1: Transient activities

To analyze the instantaneous balance, we correlate putative excitatory and inhibitory population activities in different behavioral states (sleepy rest RSS, rest RS, and movements M). We find a significantly reduced correlation (i.e., balance) during RS compared to RSS and M for monkey E, and during RS and M compared to RSS in monkey N (cf. Fig. 9A). We also observe numerous transient increases in the population spike counts (Fig. 8A). Such simultaneous peaks contribute to higher correlation values between the two neuronal populations. The prevalence of this deviations differs between behavioral states. Fig. 1 and Tab. 1 show that the distributions of population activities during M (monkey N, ns population) or both RSS and M (monkey E, both populations) are characterized by higher standard deviations than expected from higher mean values. RSS of monkey N shows lower means and slightly higher standard deviations than RS in ns population, pointing to the same conclusion. Both relations serve as footprints of an increased number of narrow peaks in population spiking during non-resting states.

Given the transient peaks in the population spike counts during M and RSS, we suspect the following relationship between balance and transients in the population activity: Whenever one of the population activities transiently increases, the other one is forced to do the same due to the recurrent coupling between putative excitatory and inhibitory neurons, yielding higher correlation value and thus more balance.

Complementary to the above discussed population activity, we now look at the firing on the level of SUs (considering 3 s time slices), shown in the top row of Fig. 2. We expect higher firing rates (mean and standard deviation) for states with more transient activities (M&RSS) which show an increased balance in the population activities. For monkey E, the reduced balance in RS compared to M (cf. Fig. 9A) coincides with a significant average firing rate reduction in RS compared to M. However, for monkey N, the reduced balance in M compared to RSS (cf. Fig. 9A) coincides with a significant firing rate increase in M compared to RSS. Since SU firing rate cannot be directly related to instantaneous balance, we examine another feature of firing, namely its regularity. The bottom row of Fig. 2 compares the results obtained for two different regularity measures, CV and CV2: only CV2 accounts

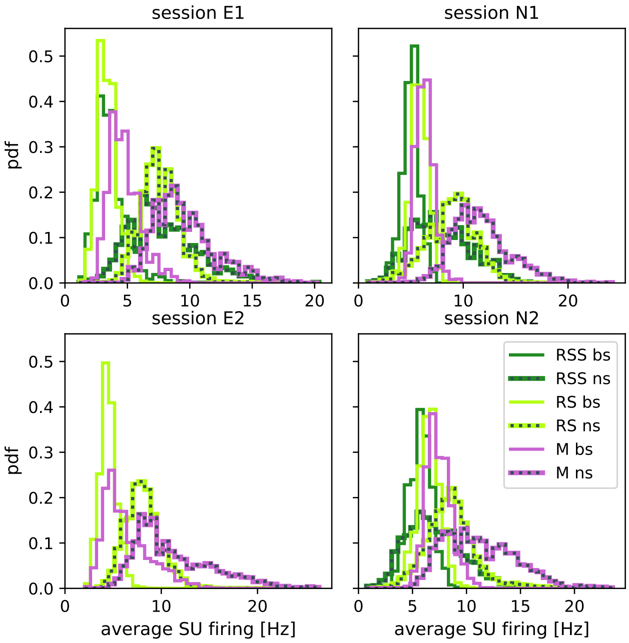

Fig. 1. Population spiking in REST recordings. Distributions of the population spiking activities calculated in 100 ms bins, separately for each REST session (left column—monkey E, right—monkey N, colors indicate behavioral states, solid lines—broad-spiking, dashed lines—narrow-spiking). Notice different ranges of the x axes.

Table 1. Mean values and standard deviations of distributions of population spiking activities visualized in Fig. 1.

Session	RSS	RS	M
E1	3.67 ± 1.23	3.36 ± 0.77	4.76 ± 1.28
	7.81 ± 2.75	7.56 ± 1.54	9.56 ± 2.49
E2		4.50 ± 0.88	6.37 ± 2.38
		8.10 ± 1.84	11.31 ± 4.06
N1	4.96 ± 0.86	5.79 ± 0.86	6.30 ± 0.85
	7.88 ± 2.91	8.85 ± 2.29	11.68 ± 2.74
N2	5.71 ± 1.10	6.52 ± 1.07	7.43 ± 1.14
	6.92 ± 2.53	8.52 ± 2.37	11.18 ± 3.34

First line per session: broad-spiking, second line: narrow-spiking population.

for transient firing rate changes which typically yield erroneously high CV values (Ponce-Alvarez et al., 2010; Voges & Perrinet, 2010). Thus, a significant difference between CV and CV2 suggests the presence of such transient firing rate changes. Indeed, we observe significantly higher CV during RSS and M. Obviously, this is no proof but only an indication for transient changes in the firing rates on the level of SU firing during movements and sleepy rest.

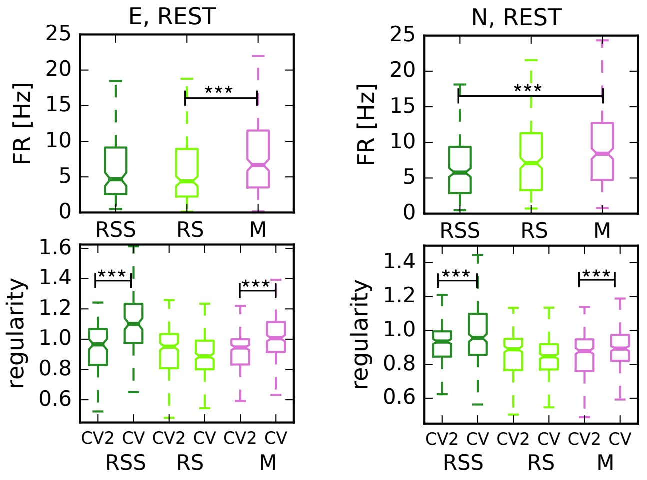

Fig. 2. Transient activities in REST recordings. Box plots of average firing rates (top row) and two different regularity measures (bottom row) of the three states defined for REST recordings (RSS in dark green, RS in light green, and M in magenta) of monkey E (left) and monkey N (right). CV2 and CV characterize the (ir-)regularity in spiking, but only CV2 accounts for transient changes in the firing rates which typically yield misleadingly high CV values. In both monkeys, CV yields significantly higher values than CV2 during M and RSS states but not during RS.

References

Ponce-Alvarez A, Kilavik B, Riehle A. 2010. Comparison of local measures of spike time irregularity and relating variability to firing rate in motor cortical neurons. J Comput Neurosci. 29:351.

Voges N, Perrinet L. 2010. Phase space analysis of networks based on biologically realistic parameters. J Physiol Paris. 104:51–60.
