# Supplementary material for: On the Complexity of Resting State Spiking Activity in Monkey Motor Cortex
Source: Cereb Cortex Commun. 2021 May 18;2(3):tgab033. doi: 10.1093/texcom/tgab033 (PMC8271144; doi:10.1093/texcom/tgab033)
Supplement: Resting_state_dynamics_S2_REVIEWED_ACCEPTED_tgab033 [file resting_state_dynamics_s2_reviewed_accepted_tgab033.pdf]

Supplement 2: Spatial activity distribution

The participation ratio quantifies the dimensionality in the network activity space. One could ask how this relates to the distribution of neuronal activity in physical space. In analogy to large-scale resting state studies which find widely distributed networks of brain areas that are particularly active during rest (Biswal et al., 1995; Raichle, 2009; Deco et al., 2011), we estimated the spatial spread of active SUs in the different behavioral states of the REST recordings. To this end, we calculated the average spatial distance from each active SU to the center of mass of the spiking activity during sleepy rest (RSS), rest (RS), and movements (M). An *active* SU emitted at least one spike during the respective 3 s slice; the center of mass is given by the average coordinates of all active SUs. We thus characterized the mean spatial spread of the activity around the center of mass in each behavioral state.

Figure 1 (below) shows the results obtained for our four REST sessions. The distinct scales on the y-axis are a result of the different implantations of the Utah arrays in the two monkeys (number and placement of active versus inactive electrodes), see Riehle et al. (2018). The differences in the spatial confinement of active SUs were small but consistent across sessions and monkeys. For monkey E, we find that the activity during RS exhibits a higher spatial spread than during M, even if the difference is only weakly significant ($p < 0.01$ in session E1 and $p < 0.05$ in session E2). In monkey N, only the second session shows a significant difference, namely a larger spatial spread in RS compared to M ($p < 0.01$). In summary, we show a tendency of the SU activity during rest to be distributed over a larger spatial region than during movement, which may relate to higher dimensionality quantified by PR.

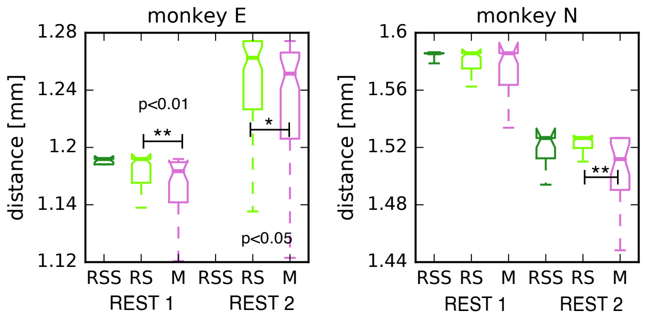

Fig. 1. Spatial arrangement of active SUs on the Utah array. Box plots of the radial spatial distance to the center of mass for the two REST recording sessions of monkey E (left panel) and N (right panel).

References

Biswal B, Zerrin YF, Haughton VM, Hyde JS. 1995. Functional connectivity in the motor cortex of resting human brain using echo-planar MRI. Magn Reson Med. 34:537–541.

Deco G, Jirsa VK, McIntosh AR. 2011. Emerging concepts for the dynamical organization of resting-state activity in the brain. Nat Rev Neurosci. 12:43–56.

Raichle ME. 2009. A paradigm shift in functional brain imaging. J Neurosci. 29:12729–12734.

Riehle A, Brochier T, Nawrot M, Grün S. 2018. Behavioral context determines network state and variability dynamics in monkey motor cortex. Front Neural Circuits. 12.
