# Supplementary material for: On the Complexity of Resting State Spiking Activity in Monkey Motor Cortex
Source: Cereb Cortex Commun. 2021 May 18;2(3):tgab033. doi: 10.1093/texcom/tgab033 (PMC8271144; doi:10.1093/texcom/tgab033)
Supplement: Resting_state_dynamics_S3_REVIEWED_ACCEPTED_tgab033 [file resting_state_dynamics_s3_reviewed_accepted_tgab033.pdf]

Supplement 3: Relation between dimensionality and number of considered neurons

We use the participation ratio (PR) to quantify the dimensionality of spiking activity across many recording sessions. However, as for every dimensionality measure, the range of possible values depends on the number of data channels considered (in our case: number of recorded single units—SUs). Therefore, the obtained values need to be corrected before any comparisons of data sets. A straightforward operation which equalizes the ranges of possible values could be a normalization by the number of SUs. However, this procedure can be adequate only if the relation between the PR and the number of SUs is linear. We test this relation within a single session by calculating PR for various subsamples of available SUs, as outlined below.

First, we define the maximum subsample size as the minimum number of SUs present in any single recording. It turns out to be 89, which is the number of SUs in an R2G session e161215-001. This choice allows us to explore the range of subsample sizes available for all our data, even if we perform this test only for REST recordings.

Second, the data is preprocessed as described in Sec. Materials and Methods: Covariances and dimensionality. For every data slice and every subsample size, we randomly pick the corresponding number of SUs and calculate the PR. This step is repeated 100 times, since different choices of SUs may lead to different PR values. We start with a subsample size of 5 and increase it in steps of 5. Finally, for each subsample size, we average PRs across repetitions for each data slice and compute the distribution of the average PR values across data slices (Fig. 1 and 2 below).

Looking at the result for the movement state of monkey N, one would notice a hint of PR saturation: At some point incorporating more SUs into the PR calculation does not affect the median of the distribution. This may suggest that, given the typical numbers of SUs we record (i.e. 120 SUs), PR values from different recording sessions could be directly compared without any normalization.

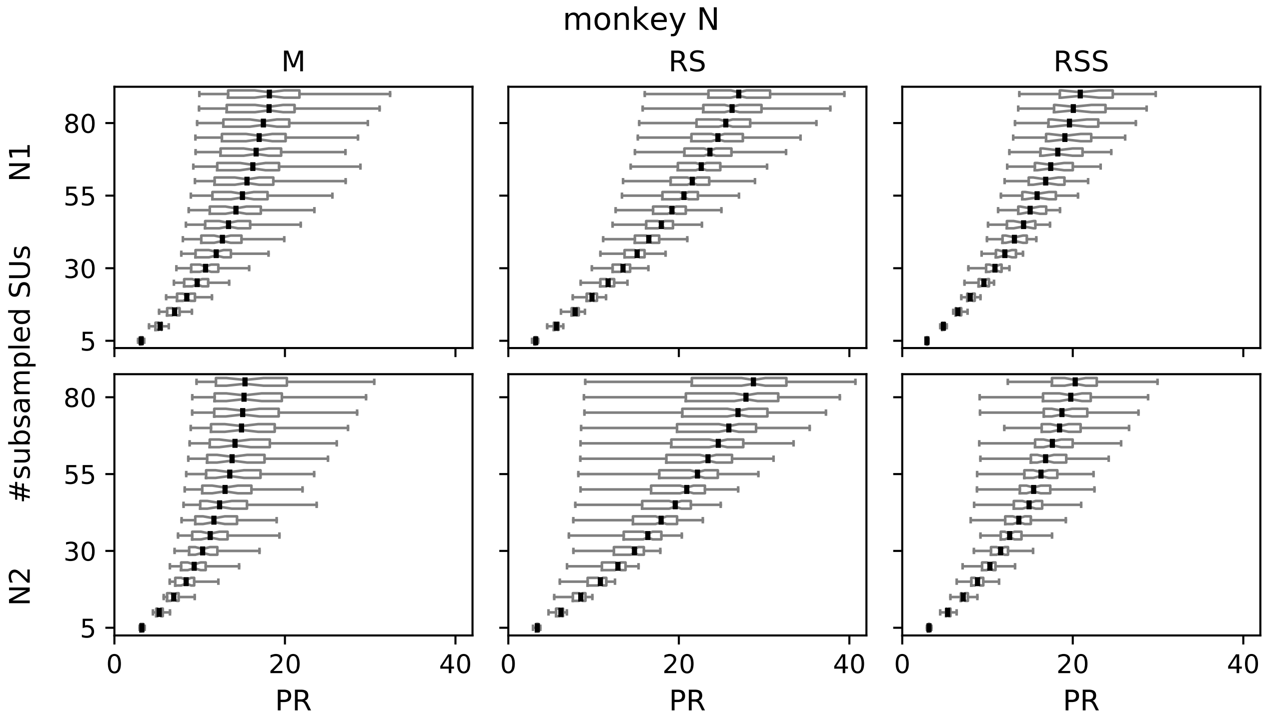

Fig. 1. Dependence of estimated participation ratio on the number of single units (monkey N). Each panel shows the distributions of PR values (x axis) obtained for different numbers of subsampled SUs (y axis) during a particular behavioral state (indicated on top) in sessions N1 (top) and N2 (bottom). The line in the center of each box represents the median, box areas represent the inter-quartile range, and the whiskers indicate minimum and maximum of the distribution, the same as for the box plots in the main text.

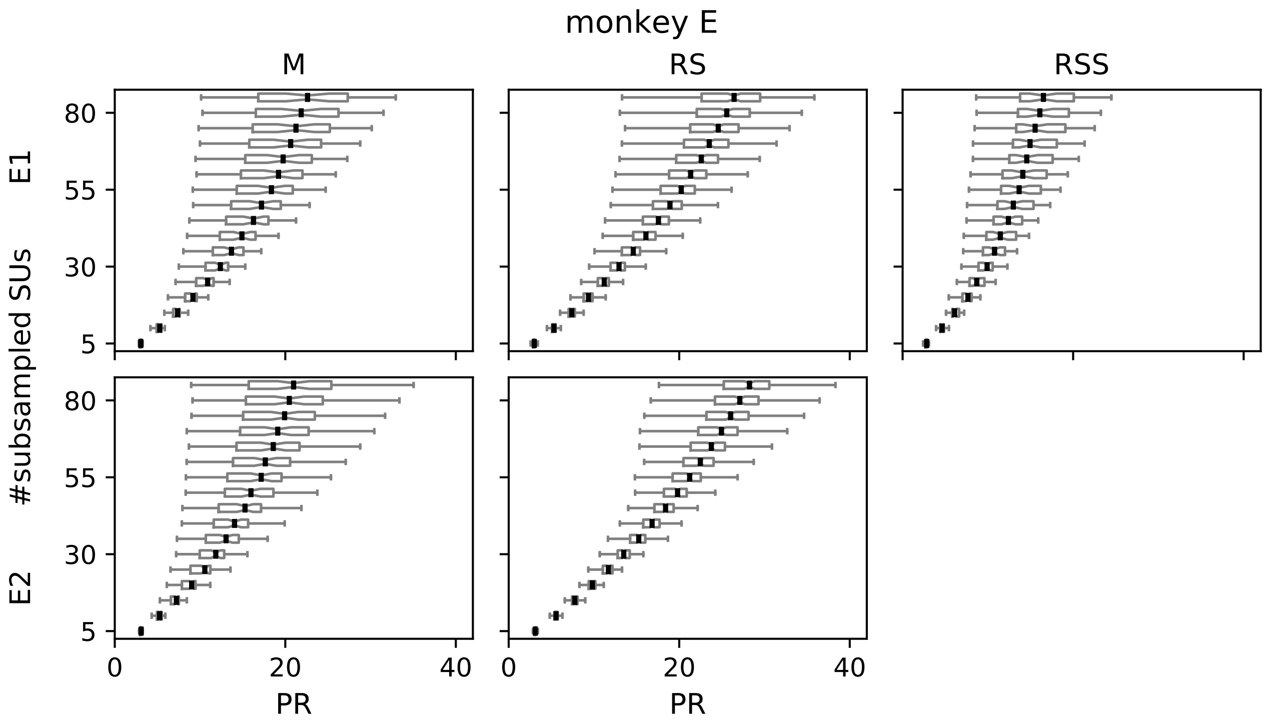

Fig. 2. Dependence of estimated participation ratio on the number of single units (monkey E). In analogy to the previous figure, sessions E1 (top) and E2 (bottom).

However, the saturation effect in monkey N is absent in rest and sleepy rest. For monkey E the results are similar, though the saturation is less pronounced even during movements.

The lack of either linear relation of PR to the number of SUs or PR saturation implies that, for our purpose, the only reliable way of comparison between sessions is to subsample the data so as to always calculate the PR based on an equal number of SUs. Of course, a particular choice of specific groups of SUs may affect the obtained values. Thus, for the results presented in the main text random subsampling was repeated 100 times and the average PR value was used.
