# Supplementary material for: On the Complexity of Resting State Spiking Activity in Monkey Motor Cortex
Source: Cereb Cortex Commun. 2021 May 18;2(3):tgab033. doi: 10.1093/texcom/tgab033 (PMC8271144; doi:10.1093/texcom/tgab033)
Supplement: Resting_state_dynamics_S4_REVIEWED_ACCEPTED_tgab033 [file resting_state_dynamics_s4_reviewed_accepted_tgab033.pdf]

Supplement 4: External resources

The preprocessed data and Python scripts reproducing figures from this publication can be found in a public repository at:

`https://doi.org/10.5281/zenodo.4696024`.
